# Supplementary material for: SERS Investigation on Oligopeptides Used as Biomimetic Coatings for Medical Devices
Source: Biomolecules. 2021 Jun 29;11(7):959. doi: 10.3390/biom11070959 (PMC8301923; doi:10.3390/biom11070959)
Supplement: Supplementary file 1 [file biomolecules-11-00959-s001.zip › biomolecules-1219361-supplementary.pdf]

Supplementary Materials

SERS investigation on oligopeptides used as biomimetic coatings for medical devices

Michele Di Foggia [1,*], Vitaliano Tugnoli [1], Stefano Ottani [2], Monica Dettin [3], Annj Zamuner [3], Santiago Sanchez-Cortes [4], Daniele Cesini [5] and Armida Torreggiani [2]

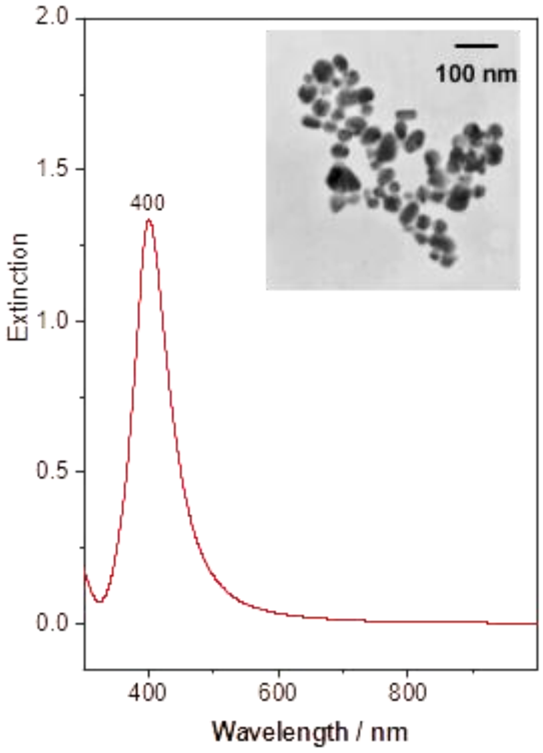

Figure S1: UV-vis spectrum and a TEM image (in the inset) of the silver colloid used in the study.

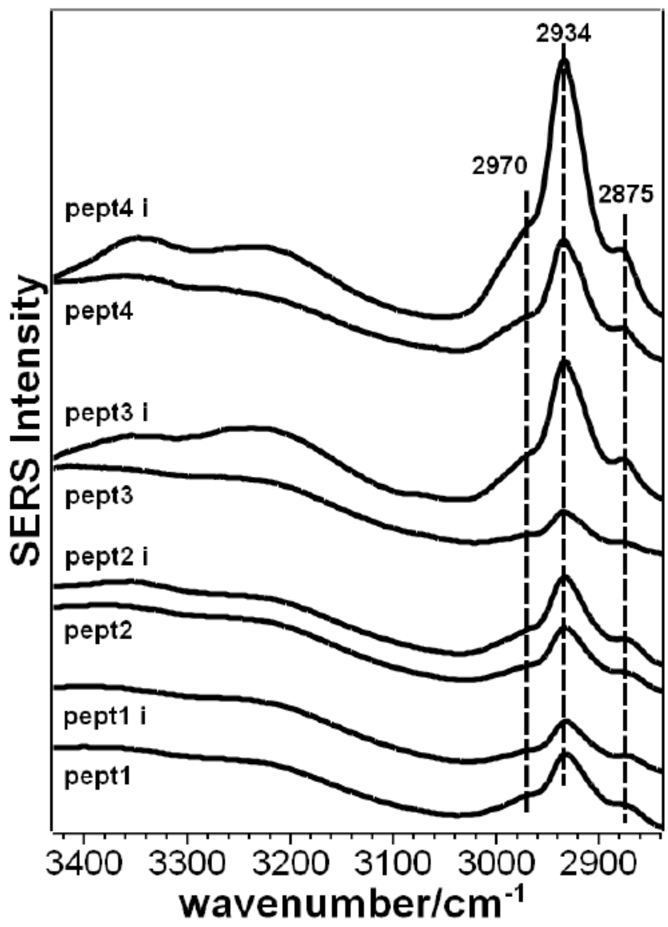

Figure S2: SERS spectra of the examined peptides before and after ${}^{\bullet}OH$ radical attack (the latter are indicated with the letter "i", indicating the experimental treatment –irradiation-) in the 3450-2800 cm^{-1} spectral range.

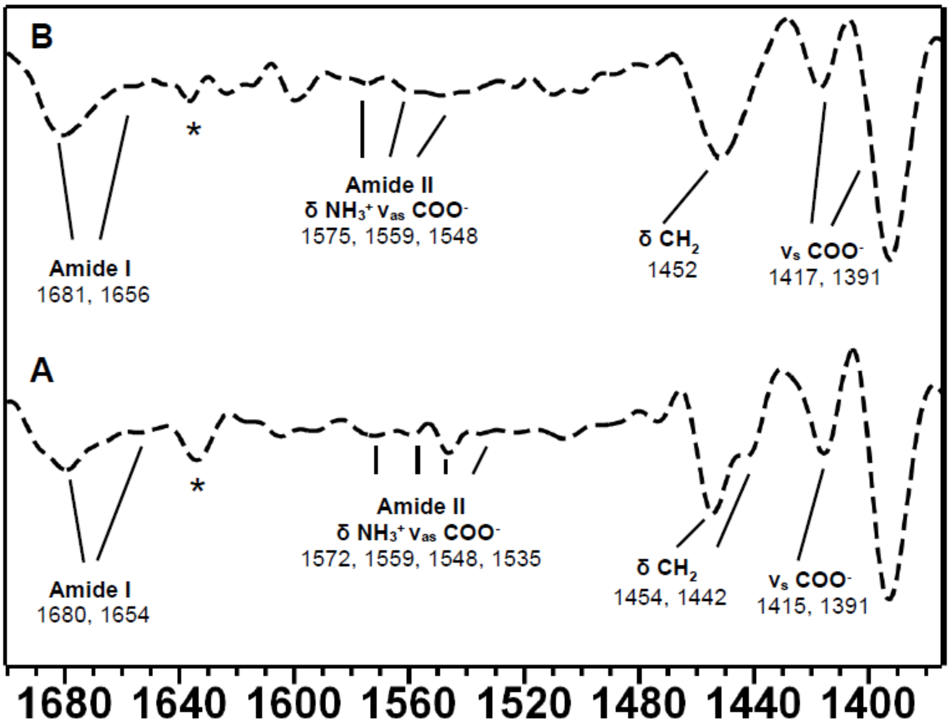

Figure S3: Second derivative SERS spectra of Pept4 before (A) and after ·OH radical attack (B) in the 1700-1350 cm^{-1} spectral range. Asterisks (*) were used to indicate the band attributed to FMOC or other contaminants.

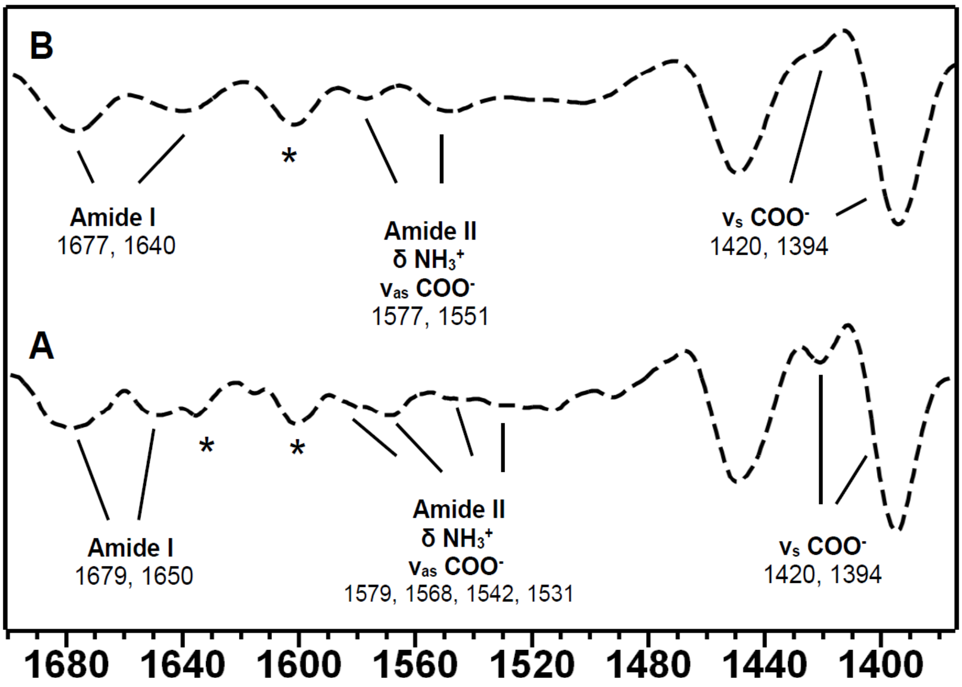

Figure S4: Second derivative SERS spectra of Pept3 before (A) and after $^{\bullet}$OH radical attack (B) in the 1700-1350 cm^{-1} spectral range. Asterisks (*) were used to indicate the bands attributed to FMOC or other contaminants.

Table S1. Interpretation of Raman SERS Spectrum of Oligopeptide $(AlaAsp)_2(AlaLys)_2$ (Ag_2/-COO¯).

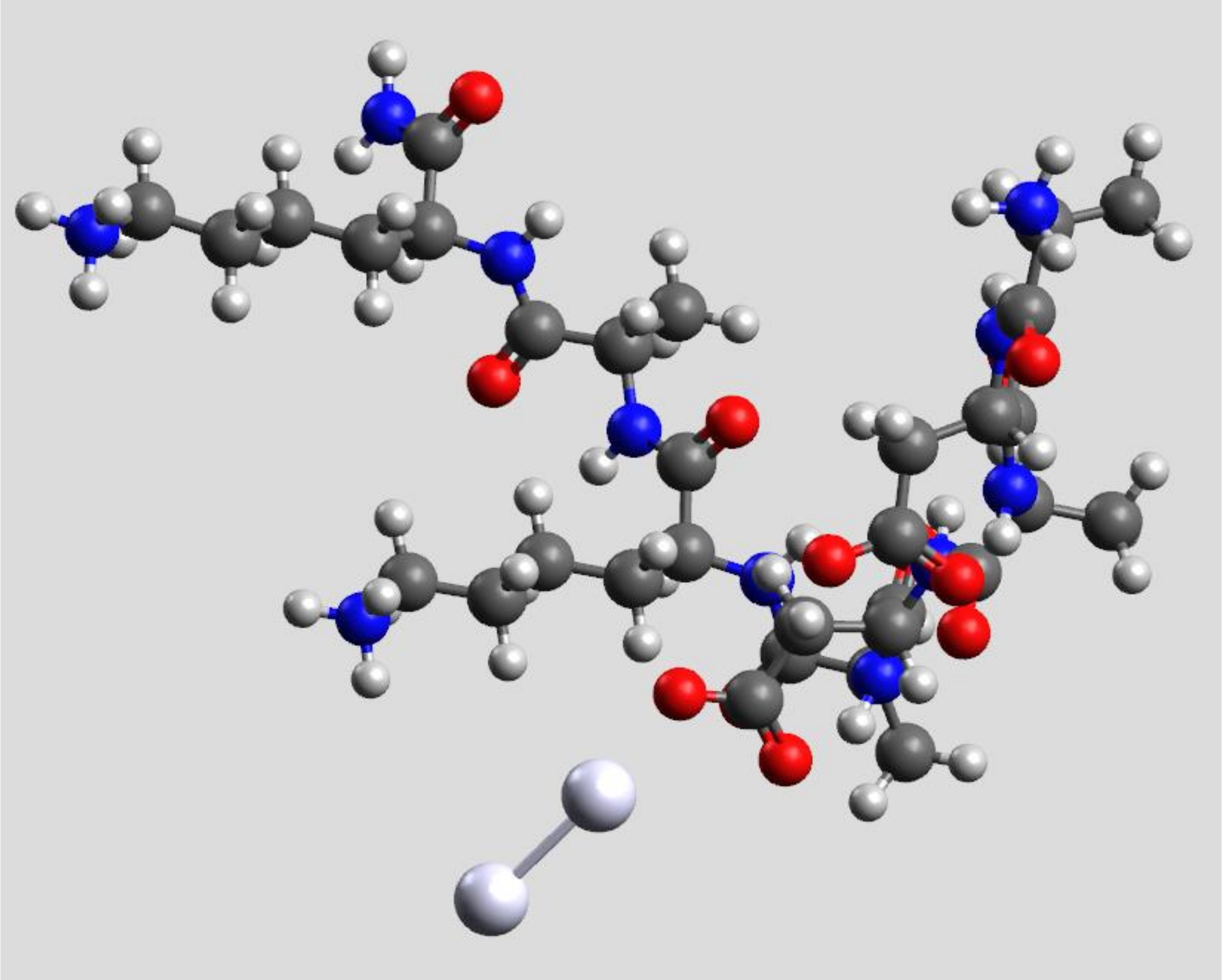

Table reports experimental frequencies (ν_{exp}, cm^{-1}), calculated frequencies (ν_{calc}, cm^{-1}), scaled frequencies ($\nu_{scaled} = 0.9306*\nu_{calc} + 34.32$; scaling straightline), percent experimental Raman Intensities (AR_{exp}), percent theoretical Raman Intensities (AR_{calc}), PED% (sxx-PED%, where sxx is the xxth internal coordinate) and mode Interpretation (ν = stretching, β = bending, τ = torsion, *out* = out-of-plane). Coalescence of several theoretical frequencies into a scaled frequency is the result of the PED fitting procedure.

ν_{exp}	AR_{exp} %	ν_{scaled}	AR_{calc}%	ν_{calc}	PED%	Interpretation
2972	29.8	2972	16.1	3616	s2-100	ν(NH)
				3157	s48-91	ν(CH)
				3156	s27-76 s28-17	ν(CH)
		2968	8.7	3603	s1-100	ν(NH)
				3153	s27-21 s28-72	ν(CH)
		2979	34.2	3621	s4-96	ν(NH)
				3167	s35-94	ν(CH)

ν_{exp}	AR_{exp} %	ν_{scaled}	AR_{calc}%	ν_{calc}	PED%	Interpretation
				3165	s19-13 s20-86	ν(CH)
				3164	s47-97	ν(CH)
				3161	s34-93	ν(CH)
		2961	13.2	3595	s6-99	ν(NH)
				3145	s19-86 s20-13	ν(CH)
		2993	18.3	3624	s3-96	ν(NH)
				3181	s44-99	ν(CH)
				3180	s57-98	ν(CH)
				3178	s21-23 s22-75	ν(CH)
2932	100.0	2931	84.6	3575	s16-100	ν(NH)
				3573	s18-99	ν(NH)
				3570	s14-91	ν(NH)
				3567	s12-100	ν(NH)
				3119	s30-77 s31-22	ν(CH)
				3116	s37-89	ν(CH)
				3114	s45-99	ν(CH)
		2940	10.7	3578	s13-90	ν(NH)
				3123	s46-95	ν(CH)
		2947	30.8	3582	s9-97	ν(NH)
				3131	s30-22 s31-77	ν(CH)
				3130	s26-88 s28-10	ν(CH)
				3129	s24-92	ν(CH)
				3129	s50-90	ν(CH)
		2906	63.7	3466	s11-99	ν(NH)
				3087	s53-97	ν(CH)
				3085	s42-87	ν(CH)
		2923	0.2	3551	s7-96	ν(NH)
				3105	s51-93	ν(CH)
		2917	0.6	3477	s17-99	ν(NH)
				3475	s15-96	ν(NH)
				3098	s40-87	ν(CH)
2868	26.8	2866	100.0	3045	s41-78 s43-13	ν(CH)
				3045	s54-92	ν(CH)
				3042	s41-14 s43-84	ν(CH)
		2877	68.4	3057	s29-98	ν(CH)
				3055	s52-89	ν(CH)

ν_{exp}	AR_{exp} %	ν_{scaled}	AR_{calc}%	ν_{calc}	PED%	Interpretation
				3053	s39-93	ν(CH)
				3052	s56-90	ν(CH)
		2884	25.8	3062	s49-98	ν(CH)
		2890	46.9	3069	s36-97	ν(CH)
				3068	s21-76 s22-23	ν(CH)
		2896	42.2	3455	s8-98	ν(NH)
				3075	s32-99	ν(CH)
				3074	s25-98	ν(CH)
2781	2.4	not explained				
2742	3.2	out of range				
1677	21.7	1601	7.4	1684	s60-79	ν(OC)
				1682	s73-73	ν(OC)
1648	23.4	1637	21.5	1722	s63-63	ν(OC)
				1721	s66-67	ν(OC)
		1628	1.3	1713	s59-73	ν(OC)
				1711	s64-70	ν(OC)
		1614	0.5	1698	s65-75	ν(OC)
		1609	0.8	1692	s61-67	ν(OC)
1556	8.1	1558	1.3	1637	s132-76 s245-22	β(HNH)+ τ(HNCC)
		1567	2.3	1649	s129-82 s241-14	β(HNH)+*out*(NHCH)
				1647	s131-76 s243-22	β(HNH)+ τ(HNCC)
		1546	1.1	1625	s123-78 s235-13	β(HNH)+*out*(NHCH)
		1576	6.1	1658	s121-82 s236-13	β(HNH)+*out*(NHCH)
				1657	s128-72 s240-22	β(HNH)+ τ(HNCC)
1534	6.6	1537	2.2	1615	s68-39 s74-15 s125-28	ν(OC)+ ν(NC)+β(HNC)
		1541	1.9	1619	s67-60 s124-19	ν(NC)+β(HNC)
		1528	0.6	1605	s119-79	β(HNH)
1512	3.8	1509	0.9	1585	s62-29 s124-17	ν(NC)+β(HNC)
		1518	1.9	1595	s71-26 s115-31	ν(NC)+β(HNC)
				1593	s68-42 s74-11 s125-14	ν(OC)+ ν(NC)+β(HNC)
				1592	s75-10 s76-22 s117-12 s118-23	ν(NC)+β(HNC)
		1504	1.2	1579	s75-21 s76-10 s117-16 s118-14	ν(NC)+β(HNC)
		1496	2.0	1570	s72-27 s116-25	ν(NC)+β(HNC)

ν_{exp}	AR_{exp} %	ν_{scaled}	AR_{calc}%	ν_{calc}	PED%	Interpretation
		1483	1.7	1557	s78-30 s126-31	ν(NC)+β(HNC)
1450	15.0	1452	0.2	1523	s130-99	β(HNH)
		1462	0.9	1535	s127-95	β(HNH)
		1427	0.5	1497	s122-87	β(HNH)
		1419	0.9	1488	s157-68	β(HCH)
				1488	s170-73	β(HCH)
1391	21.2	1387	39.9	1462	s143-56 s255-11	β(HCH)+*out*(CHCH)
				1461	s161-59	β(HCH)
				1460	s135-53	β(HCH)
				1459	s155-62	β(HCH)
				1458	s148-12 s150-61	β(HCC)+β(HCH)
				1457	s162-60 s168-11	β(HCH)
				1455	s162-12 s168-50	β(HCH)
		1401	3.9	1470	s136-58 s249-10	β(HCH)+*out*(CHCH)
				1468	s172-20	β(HCH)
		1375	9.6	1442	s159-13 s272-47	β(HCH)+τ(HCCN)
				1441	s69-63	ν(OC)
				1440	s166-10 s172-16 s284-11 s285-24	β(HCH)+τ(HCCH)
		1381	1.8	1447	s166-48	β(HCH)
		1407	1.9	1476	s166-10 s172-39	β(HCH)
				1474	s159-67	β(HCH)
		1362	7.9	1426	s140-10 s143-13	β(HCC)+β(HCH)
1331	12.3	1331	1.1	1393	s163-71	β(HCH)
		1324	2.5	1388	s139-15 s246-12	β(HCH)+τ(HCCN)
				1386	s141-89	β(HCH)
		1339	1.2	1403	s134-72	β(HCH)
				1401	s261-68	*out*(CHHH)
		1345	5.6	1409	s139-10 s146-56	β(HCH)
				1407		
		1318	1.9	1379	s163-16 s264-16 s273-10	β(HCH)+*out*(CCNH)+*out*(CCCH)
		1311	6.1	1373	s140-14 s246-24	β(HCC)+τ(HCCN)
				1370	s282-35	τ(HCCC)
		1349	2.5	1413	s69-11 s139-38	ν(OC)+β(HCH)
				1412	s280-17 s281-29 s283-12	τ(HCCC)+τ(HCCN)
		1355	2.5	1420	s273-10	*out*(CCCH)
				1419		

ν_{exp}	AR_{exp} %	ν_{scaled}	AR_{calc}%	ν_{calc}	PED%	Interpretation
1288	11.9	1288	7.6	1348	s156-60	β(HCC)
				1346	s253-42	*out*(CCNH)
		1284	13.5	1343	s167-12 s273-23	β(HCC)+*out*(CCCH)
				1343	s167-51	β(HCC)
		1293	5.9	1354	s264-13	*out*(CCNH)
				1352	s250-37	τ(HCCN)
				1352	s145-10 s257-35	β(HCC)+*out*(CCNH)
		1278	6.7	1339	s124-10 s133-12 s138-19 s253-17	β(HNC)+β(HCC)+*out*(CCNH)
				1336	s260-37	τ(HCCN)
				1335	s283-38	τ(HCCN)
		1302	4.1	1366	s260-15 s265-10 s269-15	τ(HCCN)+ τ(HCCC)
				1363	s171-48 s284-13 s285-17	β(HCN)+ τ(HCCH)
				1360	s158-51 s271-14	β(HCN)+ τ(HCCC)
1258	14.8	1257	1.3	1314	s152-16 s154-27 s268-10	β(HCC)+ τ(HCCC)
		1263	1.4	1320	s160-11 s165-18 s169-19	β(HCC)
		1273	1.6	1331	s147-52	β(HCN)
1238	13.1	1240	9.2	1296	s77-10 s164-30 s169-14	ν(NC)+β(HCC)
				1295	s115-10 s250-17 s252-11	β(HNC)+ τ(HCCN)+ τ(HCCO)
		1234	2.6	1289	s144-35 s259-10	β(HCN)+ τ(HCCO)
		1224	8.3	1281	s74-17 s125-13 s259-16	ν(NC)+β(HNC)+ τ(HCCO)
				1279	s266-10 s267-11	τ(HCCC)
				1278	s115-15 s252-17	β(HNC)+ τ(HCCO)
				1276	s278-44	τ(HCCC)
		1212	9.2	1265	s117-13 s118-10	β(HNC)
				1265	s115-10 s124-19 s138-20	β(HNC)+β(HCC)
1162	1.7	1151	14.4	1203	s81-33	ν(NC)
				1201	s83-17	ν(NC)
				1198	s145-47 s257-24	β(HCC)+*out*(CCNH)
				1195	s137-65 s252-11	β(HCC)+ τ(HCCO)
		1184	4.1	1238	s165-22 s169-17 s277-20	β(HCC)+ τ(HCCN)
				1235	s235-27 s248-23	*out*(NHCH)+*out*(CHCH)
				1233	s117-10 s151-10	β(HNC)+β(HCC)
		1190	5.6	1242	s78-10 s126-23	ν(NC)+β(HNC)
		1199	1.8	1252	s76-11 s118-16 s160-14	ν(NC)+β(HNC)+β(HCC)
		1193	4.3	1245	s72-14 s116-32 s140-11	ν(NC)+β(HNC)+β(HCC)

ν_{exp}	AR_{exp} %	ν_{scaled}	AR_{calc}%	ν_{calc}	PED%	Interpretation
1131	5.4	1130	0.4	1177	s85-13	ν(NC)
		1125	4.8	1172	s99-10 s225-17 s245-16	ν(NC)+β(CCC)+τ(HNCC)
				1170	s79-10	ν(NC)
		1137	0.2	1185	s262-13	*out*(CHCH)
		1118	2.3	1165	s79-11 s85-12	ν(NC)
1102	12.4	1099	5.6	1144	s120-10 s279-30	β(HNC)+τ(HCCC)
		1093	1.9	1140	s80-21 s89-11	ν(NC)+ν(CC)
				1137	s84-17	ν(NC)
		1086	4.1	1131	s110-10	ν(CC)
				1130	s80-10	ν(NC)
		1077	3.0	1121	s82-12	ν(NC)
				1120	s133-26 s236-16	β(HCC)+*out*(NHCH)
1051	8.6	1051	12.9	1093	s94-12 s140-15 s254-17	ν(CC)+β(HCC)+τ(HCCN)
				1092	s106-46	ν(CC)
				1091	s92-49 s120-12	ν(CC)+β(HNC)
		1045	7.9	1088	s104-44	ν(CC)
				1085	s160-21 s274-18	β(HCC)+τ(HCCN)
		1057	9.0	1099	s91-51	ν(CC)
		1037	5.9	1078	s96-60	ν(CC)
				1077	s88-69	ν(CC)
		1068	11.8	1113	s103-29 s276-17	ν(CC)+*out*(CHCH)
				1111	s101-30	ν(CC)
				1109	s77-11 s92-17 s120-19	ν(NC)+ν(CC)+β(HNC)
1018	8.6	1021	2.5	1060	s92-10 s107-30	ν(CC)
		1025	3.4	1064	s110-17 s148-14	ν(CC)+β(HCC)
		1032	1.5	1073	s148-15	β(HCC)
974	12.6	972	2.1	1008	s169-15 s243-13	β(HCC)+τ(HNCC)
		969	2.6	1004	s108-10 s235-20 s236-10 s249-26	ν(NC)+*out*(NHCH)+*out*(CHCH)
				1004	s89-31 s235-15 s236-17	ν(CC)+*out*(NHCH)
		981	2.9	1017	s154-23 s240-11	β(HCC)+τ(HNCC)
		994	11.6	1035	s87-60	ν(NC)
				1034	s99-54	ν(NC)
				1032	s102-11 s258-14	ν(CC)+τ(HCCO)
				1029	s100-11 s138-19 s327-13	ν(CC)+β(HCC)+*out*(OCOC)
935	18.1	936	1.1	969		

ν_{exp}	AR_{exp} %	ν_{scaled}	AR_{calc}%	ν_{calc}	PED%	Interpretation
		942	7.1	976	s255-14	*out*(CHCH)
				975	s274-13	τ(HCCN)
		928	2.4	961	s248-11	*out*(CHCH)
		921	2.0	953		
900	22.3	899	2.8	929	s103-15 s243-10 s276-12	ν(CC)+τ(HNCC)+*out*(CHCH)
		904	5.5	935	s105-58 s181-12	ν(CC)+β(OCO)
		889	6.5	919	s100-10 s175-16	ν(CC)+β(NCO)
		915	22.2	947	s112-41 s245-20	ν(CC)+τ(HNCC)
				946	s95-48	ν(CC)
		883	12.2	913	s262-15	*out*(CHCH)
				911		
		911	2.4	943	s111-29 s240-17	ν(CC)+τ(HNCC)
		872	3.1	901		
		866	6.0	895		
				894	s108-45 s248-11	ν(NC)+*out*(CHCH)
812	0.8	814	0.9	838	s98-19	ν(CC)
		794	2.1	817		
		778	0.7	799	s329-32	*out*(OCNC)
		876	1.0	904	s101-20 s254-10	ν(CC)+τ(HCCN)
757	2.8	756	1.9	776	s328-60	*out*(OCNC)
		764	3.3	784	s322-41	*out*(OCNC)
		748	5.2	770	s279-10 s284-16 s330-23	τ(HCCC)+τ(HCCH)+*out*(OCNC)
				770	s332-61	*out*(OCNC)
				769	s270-12 s323-36	*out*(CCCH)+*out*(OCNC)
				766	s329-10 s331-37	*out*(OCNC)
				763	s324-47	*out*(OCNC)
		772	1.6	793	s98-10 s330-31	ν(CC)+*out*(OCNC)
		737	1.7	755	s270-10	*out*(CCCH)
		733	1.8	751	s270-13	*out*(CCCH)
		727	0.3	744	s167-12 s280-38	β(HCC)+τ(HCCC)
683	3.2	684	2.4	698	s238-52	τ(HNCC)
		674	1.2	687	s93-10 s173-15 s237-26	ν(CC)+β(CCN)+τ(HNCC)
		694	0.9	709	s181-50	β(OCO)
		703	10.1	718	s182-43	β(OCO)
		709	1.3	725		
		716	2.0	732	s237-28	τ(HNCC)

ν_{exp}	AR_{exp} %	ν_{scaled}	AR_{calc}%	ν_{calc}	PED%	Interpretation
661	3.9	656	1.6	668	s237-14	τ(HNCC)
		670	0.3	683	s179-11	β(CCO)
		638	3.6	648	s173-11 s175-15	β(CCN)+β(NCO)
		641	1.3	652	s178-13 s199-10 s239-10	β(CCO)+β(CCC)+ τ(HNCC)
		627	1.3	637	s191-40 s337-12	β(NCO)+*out*(CCNC)
569	14.4	566	1.4	571		
		573	1.1	579	s326-18 s327-14	*out*(OCOC)
		562	4.3	567	s228-78	τ(HNCC)
				567	s326-15 s327-13	*out*(OCOC)
		591	1.6	599	s239-31	τ(HNCC)
				597	s233-29	τ(HNCC)
		600	0.5	608	s233-17 s239-27	τ(HNCC)
		604	0.7	612	s191-11 s233-29	β(NCO)+ τ(HNCC)
557	14.2	556	0.8	561	s231-85	τ(HNCC)
		551	1.2	556	s229-67	τ(HNCC)
		540	2.0	543	s229-15	τ(HNCC)
		545	0.4	549	s230-74	τ(HNCC)
		521	8.0	523	s184-41	β(CCO)
427	1.6	436	4.4	432	s202-23 s335-14	β(CCN)+*out*(CCNC)
		440	0.9	436	s333-13	*out*(CCNC)
		410	4.4	404	s180-11 s198-11	β(CCN)
		406	4.9	399	s194-10 s339-10	β(CCN)+*out*(CCNC)
		458	0.7	455	s202-14	β(CCN)
		472	2.4	470	s209-12 s224-14 s338-11	β(CCC)+β(CCN)+*out*(CCNC)
		477	2.2	475	s224-13 s334-11	β(CCN)+*out*(CCNC)
		477	2.2	475	s224-13 s334-11	β(CCN)+*out*(CCNC)

Table S2. Interpretation of Raman SERS Spectrum of Oligopeptide $(AlaAsp)_2(AlaLys)_2$ (Ag_2/-C=O in the peptidic chain).

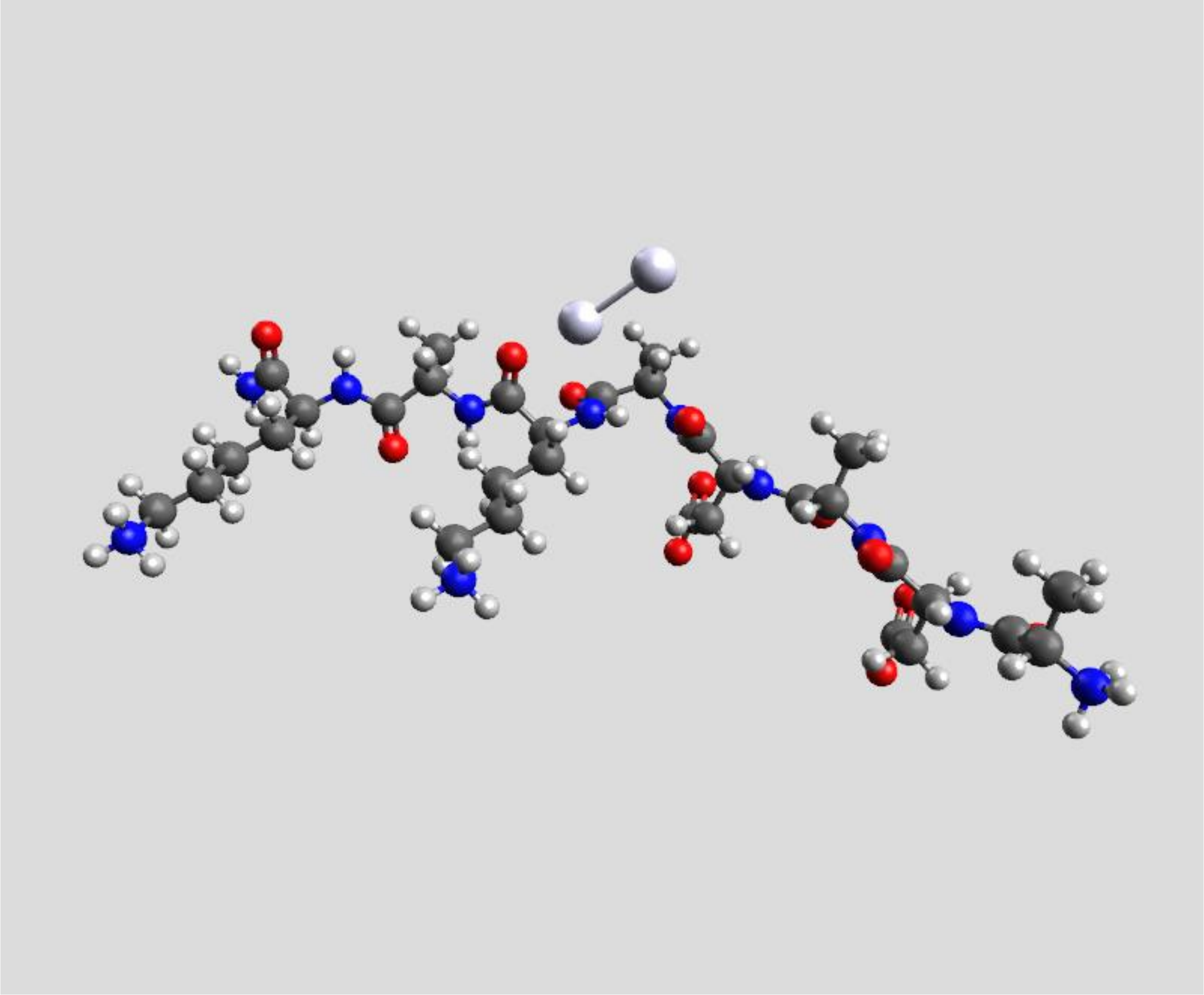

Table reports experimental frequencies (ν_{exp}, cm^{-1}), calculated frequencies (ν_{calc}, cm^{-1}), scaled frequencies ($\nu_{scaled} = 0.9311*\nu_{calc} + 31.41$; scaling straightline), percent experimental Raman Intensities (AR_{exp}), percent theoretical Raman Intensities (AR_{calc}), PED% (sxx-PED%, where sxx is the xx^{th} internal coordinate) and mode Interpretation (ν = stretching, β = bending, τ = torsion, *out* = out-of-plane). Coalescence of several theoretical frequencies into a scaled frequency is the result of the PED fitting procedure.

ν_{exp}	AR_{exp} %	ν_{scaled}	AR_{calc} %	ν_{calc}	PED%	Interpretation
2972	29.8	2973	22.4	3161	s27-99	ν(CH)
				3159	s34-14 s35-85	ν(CH)
				3158	s48-89	ν(CH)
		2968	7.3	3154	s29-95	ν(CH)
		2980	23.2	3169	s34-85 s35-14	ν(CH)
				3166	s47-100	ν(CH)

ν_{exp}	AR_{exp} %	ν_{scaled}	AR_{calc} %	ν_{calc}	PED%	Interpretation
				3165	s19-93	ν(CH)
		2964	12.1	3149	s22-91	ν(CH)
		2991	6.0	3178	s21-86	ν(CH)
		2995	10.0	3183	s55-93	ν(CH)
				3182	s43-95	ν(CH)
		2931	61.8	3116	s58-99	ν(CH)
				3115	s42-99	ν(CH)
				3115	s23-15 s25-84	ν(CH)
				3113	s49-89	ν(CH)
				3112	s50-81	ν(CH)
				3111	s44-93	ν(CH)
				3109	s30-92	ν(CH)
2932	100.0	2944	19.9	3128	s23-84 s25-15	ν(CH)
				3128	s32-92	ν(CH)
				3128	s52-88	ν(CH)
		2919	11.7	3102	s54-84	ν(CH)
				3101	s24-98	ν(CH)
				3100	s39-94	ν(CH)
		2904	50.1	3087	s40-93	ν(CH)
				3085	s56-85	ν(CH)
		2900	35.8	3081	s31-99	ν(CH)
		2868	19.2	3047	s57-91	ν(CH)
				3046	s38-78 s45-16	ν(CH)
		2874	24.9	3053	s53-94	ν(CH)
				3051	s38-14 s45-84	ν(CH)
		2864	73.2	3042	s41-93	ν(CH)
				3042	s51-88	ν(CH)
2868	26.8	2880	21.9	3059	s28-97	ν(CH)
		2883	22.6	3063	s46-96	ν(CH)
		2891	100.0	3073	s26-99	ν(CH)
				3072	s33-97	ν(CH)
				3071	s36-92	ν(CH)
				3069	s37-98	ν(CH)
				3068	s20-92	ν(CH)
2781	2.4	not explai				

ν_{exp}	AR_{exp} %	ν_{scaled}	AR_{calc} %	ν_{calc}	PED%	Interpretation
		ned				
2742	3.2	out of range				
1677	21.7	1594	3.1	1679	s61-66	ν(OC)
		1633	19.8	1722	s67-67	ν(OC)
				1719	s65-69	ν(OC)
1648	23.4			1716	s64-74	ν(OC)
		1625	0.9	1711	s62-65	ν(OC)
		1612	2.2	1697	s63-70	ν(OC)
		1602	0.9	1687	s71-67	ν(OC)
		1557	1.4	1638	s128-84 s241-14	β(HNH)+*out*(NHCH)
		1564	1.3	1646	s130-74 s244-23	β(HNH)+ τ(HNCC)
1556	8.1	1571	2.5	1654	s127-76 s240-21	β(HNH)+ τ(HNCC)
				1654	s131-75 s243-21	β(HNH)+ τ(HNCC)
		1579	1.0	1662	s121-73 s234-22	β(HNH)+ τ(HNCC)
		1586	1.2	1669	s74-64	ν(OC)
		1535	3.1	1616	s122-77 s235-16	β(HNH)+*out*(NHCH)
1534	6.6			1614	s70-66 s124-11	ν(OC)+β(HNC)
				1612	s119-80	β(HNH)
		1543	2.5	1623	s68-33 s78-18 s126-32	ν(OC)+ ν(NC)+β(HNC)
		1511	1.7	1589	s68-47 s78-10 s126-13	ν(OC)+ ν(NC)+β(HNC)
		1506	2.3	1584	s76-31 s125-30	ν(NC)+β(HNC)
				1582	s72-17 s75-11 s124-14	ν(NC)+β(HNC)
1512	3.8	1519	1.1	1598	s66-30 s118-36	ν(NC)+β(HNC)
				1597	s69-26 s70-10 s115-34	ν(NC)+ ν(OC)+β(HNC)
		1488	3.6	1565	s72-13 s75-20 s116-18	ν(NC)+β(HNC)
		1493	1.1	1570	s73-26 s117-41	ν(NC)+β(HNC)
		1450	0.3	1523	s123-94	β(HNH)
		1457	0.2	1531	s132-97	β(HNH)
1450	15.0	1462	0.2	1536	s129-96	β(HNH)
		1418	0.8	1490	s168-50 s284-37	β(HCH)+ τ(HCCC)
				1488	s159-63 s271-22	β(HCH)+ τ(HCCC)
		1392	10.5	1462	s153-61	β(HCH)
1391	21.2			1462	s136-69 s246-12 s248-12	β(HCH)+ τ(HCCN)
				1461	s166-58 s260-11	β(HCH)+ τ(HCCN)

ν_{exp}	AR_{exp} %	ν_{scaled}	AR_{calc} %	ν_{calc}	PED%	Interpretation
				1461	s162-59	β(HCH)
				1460	s142-68 s255-16	β(HCH)+*out*(CHCH)
				1459	s161-54 s274-12	β(HCH)+ τ(HCCN)
		1385	19.0	1455	s141-67 s253-14	β(HCH)+ τ(HCCN)
				1455	s170-52	β(HCH)
				1454	s146-65 s261-20	β(HCH)+ τ(HCCN)
				1454	s155-63	β(HCH)
		1399	1.5	1469	s148-19 s172-21	β(HCH)
		1373	11.6	1442	s59-62 s135-16	ν(OC)+β(HCH)
				1442	s148-14 s168-11 s281-11 s284-10	β(HCH)+ τ(HCCC)
				1441	s268-45	τ(HCCN)
				1441	s60-65 s258-12	ν(CC)+ τ(HCCO)
		1405	3.1	1476	s172-37	β(HCH)
				1475	s157-64	β(HCH)
				1474	s134-60 s138-12 s247-11	β(HCH)+ τ(HCCN)
		1379	1.4	1447	s148-21 s172-11 s281-22	β(HCH)+ τ(HCCC)
		1332	1.1	1396	s151-65	β(HCN)
		1338	5.2	1405	s139-77	β(HCH)
				1404	s149-48	β(HCH)
				1401	s150-64	β(HCH)
		1322	4.8	1387	s143-66	β(HCH)
				1386		
				1385	s163-32	β(HCH)
1331	12.3	1347	3.6	1414	s168-15 s281-14	β(HCH)+ τ(HCCC)
				1413	s59-14 s135-56	ν(OC)+β(HCH)
				1412	s271-13	τ(HCCC)
		1315	3.1	1379	s117-10 s147-31	β(HNC)+β(HCC)
		1353	3.6	1419	s60-13 s144-10	ν(CC)+β(HCN)
		1361	5.7	1429	s138-37	β(HCH)
				1426	s160-10	β(HCC)
		1286	2.4	1347	s273-26 s278-10	*out*(CCNH)+ τ(HCCC)
		1292	1.9	1354	s137-11 s250-38	β(HCC)+ τ(HCCN)
				1353	s144-11 s257-39 s262-14	β(HCN)+ τ(HCCN)
1288	11.9	1280	14.5	1342	s167-61	β(HCC)
				1341	s257-10 s262-39	τ(HCCN)
				1341	s156-47 s273-13	β(HCC)+*out*(CCNH)

ν_{exp}	AR_{exp} %	ν_{scaled}	AR_{calc} %	ν_{calc}	PED%	Interpretation
		1275	17.2	1338	s137-28 s254-12	β(HCC)+ τ(HCCN)
				1337	s278-22 s282-10	τ(HCCC)
				1336	s167-10 s266-24	β(HCC)+*out*(CCNH)
		1298	1.4	1360	s158-67 s241-11	β(HCN)+*out*(NHCH)
		1303	6.5	1366		
				1366	s171-58 s243-10	β(HCN)+ τ(HNCC)
				1365	s152-21 s271-19	β(HCC)+ τ(HCCC)
		1309	1.9	1372	s277-24	τ(HCCN)
		1259	1.4	1318	s165-33 s273-11	β(HCC)+*out*(CCNH)
1258	14.8	1265	2.1	1325	s140-15 s254-12	β(HCC)+ τ(HCCN)
		1249	2.5	1308	s154-22 s264-30	β(HCC)+*out*(CCNH)
		1239	9.1	1298	s77-10 s164-28 s165-16	ν(NC)+β(HCC)
				1296	s115-12 s252-20	β(HNC)+ τ(HCCO)
		1233	5.4	1292	s140-10 s144-18 s252-12	β(HCC)+β(HCN)+ τ(HCCO)
				1291	s269-29	τ(HCCC)
		1228	3.4	1285	s78-16 s126-10 s259-23	ν(NC)+β(HNC)+ τ(HCCO)
1238	13.1	1222	4.6	1279	s278-10 s282-25	τ(HCCC)
				1279	s115-18 s137-14 s252-36	β(HNC)+β(HCC)+ τ(HCCO)
		1213	6.6	1271	s66-11 s118-21 s282-15	ν(NC)+β(HNC)+ τ(HCCC)
				1268	s73-18 s117-16 s147-13	ν(NC)+β(HNC)+β(HCC)
		1195	11.4	1251	s125-25 s160-18	β(HNC)+β(HCC)
				1250	s75-13 s116-28 s140-15	ν(NC)+β(HNC)+β(HCC)
		1156	5.0	1208	s84-19 s85-10	ν(NC)
		1151	3.4	1203	s80-21	ν(NC)
		1145	2.3	1197	s145-41 s259-29	β(HCC)+ τ(HCCO)
1162	1.7	1185	3.4	1240	s169-31 s283-11	β(HCC)+ τ(HCCN)
				1238	s270-23	*out*(CCCH)
		1180	0.2	1233	s136-10 s235-25 s248-24	β(HCH)+*out*(NHCH)+ τ(HCCN)
		1203	3.5	1258	s72-12 s116-10 s124-28	ν(NC)+β(HNC)
		1134	2.8	1184	s82-13 s260-12	ν(NC)+ τ(HCCN)
		1127	3.6	1177	s91-12 s225-20 s244-19	ν(CC)+β(CCN)+ τ(HNCC)
1131	5.4	1141	4.9	1193	s133-64	β(HCC)
				1190	s84-13 s85-13	ν(NC)
		1121	1.1	1171	s79-15	ν(NC)
		1116	0.7	1164	s82-11	ν(NC)

ν_{exp}	AR_{exp} %	ν_{scaled}	AR_{calc} %	ν_{calc}	PED%	Interpretation
		1104	3.8	1152	s152-15 s267-12 s270-16	β(HCC)+ τ(HCCC)+*out*(CCCH)
		1098	5.3	1146	s120-12 s283-26	β(HNC)+ τ(HCCN)
		1094	0.7	1141	s81-21 s89-12	ν(NC)+ ν(CC)
1102	12.4	1089	2.6	1136	s83-12 s247-14	ν(NC)+ τ(HCCN)
				1135	s83-16 s234-10	ν(NC)+ τ(HNCC)
		1079	0.9	1125	s85-12	ν(NC)
		1082	0.4	1128	s79-12 s89-12	ν(NC)+ ν(CC)
		1052	5.4	1096	s87-57	ν(CC)
		1049	4.7	1093	s88-49	ν(CC)
				1092	s101-14 s105-26	ν(CC)
		1042	12.9	1088	s101-24 s105-14	ν(CC)
1051	8.6			1087	s160-11 s274-16	β(HCC)+ τ(HCCN)
				1084	s140-22	β(HCC)
		1066	9.9	1112	s106-22 s120-11	ν(CC)+β(HNC)
				1111	s103-36 s253-11	ν(CC)+ τ(HCCN)
				1110	s106-19 s120-11	ν(CC)+β(HNC)
		1015	1.1	1057	s109-18 s261-15	ν(CC)+ τ(HCCN)
		1023	2.3	1065	s112-38	ν(CC)
1018	8.6	1033	8.0	1080	s86-52	ν(CC)
				1077	s96-63	ν(CC)
				1075	s90-13 s261-10	ν(CC)+ τ(HCCN)
		999	0.9	1039	s94-63	ν(NC)
		973	4.3	1014	s89-30 s234-21 s247-20	ν(CC)+ τ(HNCC)+ τ(HCCN)
				1011	s169-14 s243-11	β(HCC)+ τ(HNCC)
				1011	s154-10 s241-15 s267-10	β(HCC)+*out*(NHCH)+ τ(HCCC)
974	12.6	989	5.3	1029	s258-16 s323-12	τ(HCCO)+*out*(OCOC)
		962	0.9	1000	s108-10 s235-34 s246-22	ν(NC)+*out*(NHCH)+ τ(HCCN)
		993	2.8	1033	s107-63	ν(NC)
				1032	s102-10 s137-10 s325-10	ν(CC)+β(HCC)+*out*(OCOC)
		940	3.5	976	s95-13 s275-23	ν(CC)+*out*(CHCH)
				973	s98-12 s255-18	ν(CC)+*out*(CHCH)
935	18.1	930	1.7	965	s111-11	ν(CC)
		927	0.9	962	s111-10	ν(CC)
		921	5.1	957	s91-32 s244-13	ν(CC)+ τ(HNCC)
				955	s91-18	ν(CC)

ν_{exp}	AR_{exp} %	ν_{scaled}	AR_{calc} %	ν_{calc}	PED%	Interpretation
		900	12.9	934	s92-54 s181-11	ν(CC)+β(OCO)
				933	s93-59 s182-11	ν(CC)+β(OCO)
				931	s106-15 s243-10	ν(CC)+ τ(HNCC)
		890	6.8	922	s175-11 s251-16	β(NCO)+*out*(CCCH)
		879	10.6	910	s103-11	ν(CC)
900	22.3	914	1.4	948	s111-11	ν(CC)
		862	8.7	894		
				891	s108-51	ν(NC)
		871	1.9	904		
				902	s103-13	ν(CC)
				900		
		812	0.8	839	s100-24 s280-14	ν(CC)+ τ(HCCC)
812	0.8	795	2.8	820	s265-11 s328-33	*out*(CCCH)+*out*(OCNC)
		788	0.4	812	s265-46 s328-11	*out*(CCCH)+*out*(OCNC)
		762	3.0	784	s326-33	*out*(OCNC)
		758	0.3	780	s229-14 s337-37	τ(HNCC)+*out*(OCNC)
		749	4.9	772	s280-10 s285-31 s329-23	τ(HCCC)+ τ(HCCN)+*out*(OCNC)
				771	s330-47	*out*(OCNC)
				771	s322-48	*out*(OCNC)
757	2.8	771	1.0	794	s100-10 s329-33	ν(CC)+*out*(OCNC)
		739	1.6	761	s324-42	*out*(OCNC)
				760	s229-38	τ(HNCC)
				758	s272-56	*out*(CCCH)
		746	0.1	767	s327-51	*out*(OCNC)
		724	0.3	744	s156-13 s279-59	β(HCC)+ τ(HCCC)
		688	0.8	705	s181-13	β(OCO)
		695	2.7	713	s182-40	β(OCO)
683	3.2			710	s181-30	β(OCO)
		704	1.3	722	s99-12 s265-15 s267-11	ν(CC)+*out*(CCCH)+ τ(HCCC)
		716	2.0	736	s326-17	*out*(OCNC)
		661	3.9	676		
		666	5.1	683	s221-13 s228-51	β(CCN)+ τ(HNCC)
661	3.9			681	s230-11	τ(HNCC)
		648	5.0	662	s174-11 s230-18	β(CCC)+ τ(HNCC)
		634	1.0	647	s176-38	β(NCO)

ν_{exp}	AR_{exp} %	ν_{scaled}	AR_{calc} %	ν_{calc}	PED%	Interpretation
		627	1.9	639	s178-35 s233-14	*β*(NCO)+ *τ*(HNCC)
569	14.4	567	1.9	575	s231-42	*τ*(HNCC)
		570	0.3	578	s323-17 s325-20	*out*(OCOC)
		590	2.3	602	s205-17	*β*(CCN)
				600	s188-25 s233-14	*β*(CCN)+ *τ*(HNCC)
		613	7.8	625	s230-43 s233-13	*τ*(HNCC)
				623	s178-10 s233-41	*β*(NCO)+ *τ*(HNCC)
557	14.2	560	3.7	570	s237-81	*τ*(HNCC)
				569	s238-82	*τ*(HNCC)
				568	s323-17 s325-18	*out*(OCOC)
		546	1.2	553	s231-38	*τ*(HNCC)
		541	0.9	548	s239-38	*τ*(HNCC)
		529	0.6	535	s239-47	*τ*(HNCC)
		512	1.6	516	s332-13	*out*(CCNC)
427	1.6	437	4.9	437	s202-11 s332-14	*β*(CCN)+*out*(CCNC)
				435	s202-30	*β*(CCN)
		414	4.3	411	s198-12 s212-10	*β*(CCN)
		450	1.3	449	s207-23 s334-15	*β*(CCC)+*out*(CCNC)
		401	2.3	397	s339-20	*out*(CCNC)
		393	2.9	388		
		384	1.6	379	s193-44	*β*(CCN)
		462	0.5	463	s202-12 s335-15	*β*(CCN)+*out*(CCNC)
		470	0.8	471	s224-22	*β*(CCN)
		476	0.4	477	s209-57	*β*(CCN)
		495	1.6	498	s183-35 s333-18	*β*(CCO)+*out*(CCNC)
		500	2.6	503	s184-32 s331-14	*β*(CCO)+*out*(CCNC)

Table S3. Interpretation of Raman SERS Spectrum of Oligopeptide $(AlaAsp)_2(AlaLys)_2$

(Ag_2/-C=O in the Peptidic Chain, 2nd Setting).

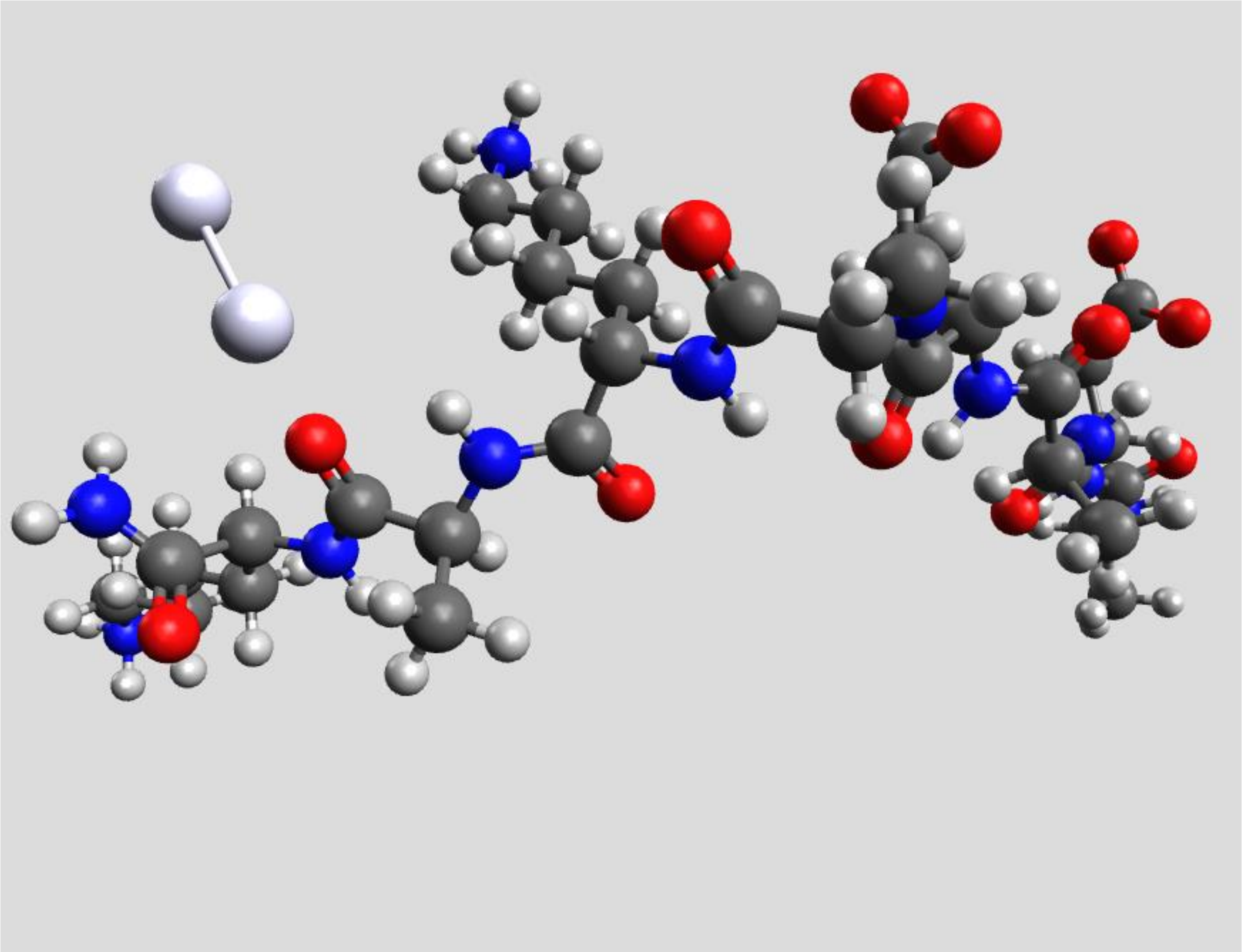

Table reports experimental frequencies (ν_{exp}, cm^{-1}), calculated frequencies (ν_{calc}, cm^{-1}), scaled frequencies ($\nu_{scaled} = 0.9309*\nu_{calc} + 31.92$; scaling straightline), percent experimental Raman Intensities (AR_{exp}), percent theoretical Raman Intensities (AR_{calc}), PED% (sxx-PED%, where sxx is the xxth internal coordinate) and mode Interpretation (ν = stretching, β = bending, τ = torsion, *out* = out-of-plane). Coalescence of several theoretical frequencies into a scaled frequency is the result of the PED fitting procedure.

ν_{exp}	AR_{exp} %	$\nu_{\sigma caled}$	AR_{calc} %	ν_{calc}	PED%	Interpretation
		2972	22.8	3160	s34-92	ν(CH)
				3160	s27-99	ν(CH)
				3157	s48-92	ν(CH)
2972	29.8			3154	s29-91	ν(CH)
		2979	18.5	3168	s35-92	ν(CH)
				3166	s19-16 s20-83	ν(CH)
				3164	s47-97	ν(CH)

ν_{exp}	AR_{exp} %	ν_{scaled}	AR_{calc} %	ν_{calc}	PED%	Interpretation
		2963	9.0	3148	s19-83 s20-16	ν(CH)
		2993	11.6	3183	s57-98	ν(CH)
				3180	s21-22 s22-76	ν(CH)
				3180	s44-98	ν(CH)
		2930	43.3	3115	s58-99	ν(CH)
				3115	s55-95	ν(CH)
				3113	s30-85 s31-13	ν(CH)
				3112	s45-99	ν(CH)
				3111	s40-22 s42-63	ν(CH)
				3109	s23-93	ν(CH)
		2935	7.7	3118	s46-95	ν(CH)
2932	100.0	2942	22.0	3128	s24-93	ν(CH)
				3128	s30-14 s31-85	ν(CH)
				3125	s37-87	ν(CH)
				3124	s26-93	ν(CH)
		2918	8.6	3100	s33-98	ν(CH)
		2922	0.8	3105	s51-95	ν(CH)
				3104	s38-89	ν(CH)
		2908	17.8	3089	s53-96	ν(CH)
		2868	35.0	3046	s54-80 s56-11	ν(CH)
		2863	24.6	3041	s41-96	ν(CH)
		2877	100.0	3061	s49-98	ν(CH)
				3059	s28-98	ν(CH)
				3057	s50-83 s52-14	ν(CH)
				3054	s39-89	ν(CH)
				3053	s54-10 s56-81	ν(CH)
2868	26.8			3052	s43-95	ν(CH)
				3050	s50-12 s52-73	ν(CH)
		2891	63.2	3074	s25-99	ν(CH)
				3072	s32-99	ν(CH)
				3070	s21-77 s22-23	ν(CH)
				3069	s36-97	ν(CH)
		2902	20.9	3083	s40-76 s42-20	ν(CH)
2781	2.4	not explained				

ν_{exp}	AR_{exp} %	ν_{scaled}	AR_{calc} %	ν_{calc}	PED%	Interpretation
2742	3.2	out of range				
1677	21.7	1612	0.7	1697	s66-69	ν(OC)
		1633	13.5	1721	s65-67	ν(OC)
				1718	s64-66	ν(OC)
1648	23.4	1626	4.3	1713	s61-75	ν(OC)
				1710	s59-65	ν(OC)
		1604	0.6	1688	s62-67	ν(OC)
		1562	2.9	1645	s127-71 s240-16	β(HNH)+ τ(HNCC)
				1644	s132-77 s244-13	β(HNH)+*out*(NHCH)
				1642	s131-77 s243-14	β(HNH)+ τ(HNCC)
1556	8.1	1572	1.7	1656	s122-69 s236-14	β(HNH)+ τ(HCNH)
				1654	s129-72 s241-17	β(HNH)+ τ(HNCC)
		1589	2.0	1673	s60-61 s66-10	ν(OC)
		1596	2.1	1680	s63-73	ν(OC)
		1536	4.0	1617	s121-71 s234-10 s235-10	β(HNH)+ τ(HNCC)
1534	6.6			1616	s72-59 s124-20	ν(NC)+β(HNC)
				1615	s119-84	β(HNH)
		1544	1.5	1625	s73-42 s125-35	ν(NC)+β(HNC)
		1514	1.3	1592	s75-49 s116-17	ν(OC)+β(HNC)
		1508	1.3	1588	s67-49	ν(NC)
				1586	s76-20 s117-19 s118-12	ν(NC)+β(HNC)
1512	3.8			1584	s68-28 s115-14 s116-12	ν(NC)+β(HNC)
		1499	2.1	1576	s71-35 s124-14	ν(NC)+β(HNC)
		1521	0.5	1600	s74-27 s117-12 s118-29	ν(NC)+β(HNC)
		1493	1.0	1570	s78-27 s126-35	ν(NC)+β(HNC)
		1442	0.1	1515	s130-97	β(HNH)
		1456	0.1	1529	s128-96	β(HNH)
1450	15.0	1417	1.9	1489	s172-64	β(HCH)
				1488	s159-72	β(HCH)
				1487	s123-78	β(HNH)
		1388	14.1	1468	s134-62 s249-10	β(HCH)+*out*(CHCH)
1391	21.2			1465	s135-59 s248-10	β(HCH)+*out*(CHCH)
				1465	s170-63	β(HCH)
				1464	s162-47 s275-10	β(HCH)+*out*(CHCH)

ν_{exp}	AR_{exp} %	ν_{scaled}	AR_{calc} %	ν_{calc}	PED%	Interpretation
				1463	s142-50 s255-10	β(HCH)+*out*(CHCH)
				1460	s155-66	β(HCH)
				1458	s150-57	β(HCH)
		1382	9.6	1451	s153-68	β(HCH)
				1451	s149-75 s263-10	β(HCH)+*out*(CHCH)
				1448	s77-10 s170-11 s279-13 s281-11	ν(NC)+β(HCH)+τ(HCCC)
		1404	3.5	1474	s157-71	β(HCH)
		1372	7.3	1440	s168-11 s283-39	β(HCH)+τ(HCCN)
				1440	s70-62	ν(OC)
				1439	s270-40	τ(HCCH)
				1439	s69-65	ν(OC)
		1407	1.3	1477	s166-67	β(HCH)
		1336	1.9	1401	s139-18 s160-27	β(HCH)
				1400	s139-11 s160-23 s246-10	β(HCH)+τ(HCCN)
				1399	s148-60	β(HCH)
		1322	5.3	1388	s143-57	β(HCH)
				1387	s143-19 s246-10	β(HCH)+τ(HCCN)
				1385	s163-27	β(HCC)
1331	12.3	1343	4.4	1409	s146-59	β(HCH)
				1408	s266-16	τ(HCCC)
		1349	1.9	1416	s70-10 s139-32	ν(OC)+β(HCH)
				1414	s165-12 s172-14 s281-19	β(HCC)+β(HCH)+τ(HCCC)
				1412	s136-62	β(HCH)
		1359	5.8	1426		
				1425		
		1287	0.7	1348	s145-11 s257-34 s260-18	β(HCC)+*out*(CCNH)+*out*(CCCH)
		1280	14.2	1345	s253-41	*out*(CCNH)
				1344	s252-25	*out*(CCNH)
				1342	s273-43	*out*(CCNH)
				1340	s169-54	β(HCC)
1288	11.9			1338	s156-25 s272-21	β(HCC)+τ(HCCH)
				1338	s137-24	β(HCC)
		1299	5.9	1364	s152-18 s264-22	β(HCC)+τ(HCCN)
				1362	s147-20	β(HCC)
				1360	s171-49 s284-13	β(HCN)+τ(HCCC)
				1359	s158-45 s240-10 s270-10 s271-12	β(HCN)+τ(HNCC)+τ(HCCH)+τ(HCCC)

ν_{exp}	AR_{exp} %	ν_{scaled}	AR_{calc} %	ν_{calc}	PED%	Interpretation
		1307	6.8	1370	s165-15 s277-11	β(HCC)+*out*(CCCH)
				1369	s246-18	τ(HCCN)
		1263	1.1	1322	s273-14 s277-39	*out*(CCNH)+*out*(CCCH)
		1272	10.3	1334	s260-42	*out*(CCCH)
1258	14.8			1332	s279-29	τ(HCCC)
				1331	s156-40 s266-21	β(HCC)+τ(HCCC)
		1248	2.3	1307	s167-52	β(HCC)
		1252	0.4	1311	s152-44	β(HCC)
		1234	3.2	1291	s73-10 s144-40	ν(NC)+β(HCN)
		1228	6.1	1286	s281-11	τ(HCCC)
				1285	s250-29	τ(HCCO)
				1284	s257-13 s259-25	*out*(CCNH)+τ(HCCO)
1238	13.1	1218	6.4	1275	s281-11	τ(HCCC)
				1274	s268-36	τ(HCCC)
				1273	s124-13 s250-14	β(HNC)+τ(HCCO)
		1211	4.0	1267		
				1266	s116-12 s140-14	β(HNC)+β(HCC)
		1156	2.3	1207	s81-37 s255-10	ν(NC)+*out*(CHCH)
		1151	4.1	1203	s84-25	ν(NC)
		1184	2.1	1237	s154-22 s269-10	β(HCC)+*out*(CCCH)
1162	1.7	1176	0.1	1229	s248-13 s249-10 s331-20	*out*(CHCH)+*out*(CCNC)
		1188	0.8	1242	s164-41 s284-10	β(HCC)+τ(HCCC)
		1194	4.6	1248	s115-39 s137-21	β(HNC)+β(HCC)
		1199	2.4	1254	s78-12 s126-23 s151-13	ν(NC)+β(HNC)+β(HCN)
		1132	0.9	1181		
		1125	2.2	1174		
1131	5.4	1142	4.6	1193	s138-45 s251-12 s252-14	β(HCC)+τ(HCCO)+*out*(CCNH)
				1193	s138-11 s145-34 s257-17	β(HCC)+*out*(CCNH)
				1191	s82-10 s262-11	ν(NC)+*out*(CHCH)
		1120	1.0	1169	s79-11	ν(NC)
		1105	1.7	1153	s101-15 s282-32	ν(CC)+τ(HCCC)
		1114	1.8	1163	s79-13	ν(NC)
1102	12.4	1091	0.7	1140	s80-22 s89-11	ν(NC)+ν(CC)
				1137	s83-10	ν(NC)
		1083	4.2	1131	s89-13 s248-11	ν(CC)+*out*(CHCH)

ν_{exp}	AR_{exp} %	ν_{scaled}	AR_{calc} %	ν_{calc}	PED%	Interpretation
				1129	s83-12	ν(NC)
1051	8.6	1053	5.4	1096	s90-50	ν(CC)
		1047	16.7	1093	s92-55	ν(CC)
				1092	s140-10 s256-11	β(HCC)+ τ(HCCN)
				1090	s102-12 s109-23	ν(CC)
				1088	s276-12	*out*(CHCH)
				1087	s102-25 s109-11	ν(CC)
		1068	4.6	1113	s105-22 s276-12	ν(CC)+*out*(CHCH)
				1112	s99-28	ν(CC)
		1036	1.5	1079	s88-58	ν(NC)
		1077	4.1	1123	s77-16 s120-25	ν(NC)+β(HNC)
		1073	1.8	1119	s82-11	ν(NC)
				1118	s133-41	β(HCC)
1018	8.6	1021	2.0	1063	s147-16 s263-21	β(HCC)+*out*(CHCH)
		1028	3.7	1072	s96-23	ν(CC)
				1069	s96-13	ν(CC)
		1032	0.9	1075	s87-60	ν(CC)
974	12.6	980	1.5	1019	s154-16 s158-11 s240-16	β(HCC)+β(HCN)+ τ(HNCC)
		964	1.9	1002	s89-29 s234-16 s248-17	ν(CC)+ τ(HNCC)+*out*(CHCH)
				1001	s167-16 s244-20 s285-14	β(HCC)+*out*(NHCH)+ τ(HCCC)
		990	6.5	1031	s97-69	ν(NC)
				1030	s98-10 s329-12	ν(CC)+*out*(OCNC)
				1029	s100-10 s258-12	ν(CC)+ τ(HCCO)
		995	1.2	1034	s106-62	ν(NC)
		954	0.6	991	s235-17 s249-28	τ(HNCC)+*out*(CHCH)
935	18.1	936	1.7	971	s275-15	*out*(CHCH)
		932	0.6	967		
		939	0.6	975	s255-15 s262-11	*out*(CHCH)
		924	2.8	958	s248-10	*out*(CHCH)
		917	1.3	951	s112-11	ν(CC)
900	22.3	900	7.6	933	s103-56 s181-10	ν(CC)+β(OCO)
				932	s104-58 s182-10	ν(CC)+β(OCO)
		888	5.0	920	s98-10 s176-16 s329-10	ν(CC)+β(NCO)+*out*(OCNC)
		908	0.8	941	s111-39 s241-21	ν(CC)+ τ(HNCC)
		912	2.3	945	s112-32 s243-18	ν(CC)+ τ(HNCC)

ν_{exp}	AR_{exp} %	ν_{scaled}	AR_{calc} %	ν_{calc}	PED%	Interpretation
		879	8.0	911		
				910		
		885	1.3	916	s107-10 s244-23	ν(CC)+*out*(NHCH)
		871	4.2	903	s99-14	ν(CC)
				901	s105-12	ν(CC)
		808	1.4	833	s107-19	ν(CC)
		792	3.0	816	s269-16 s327-11	*out*(CCCH)+*out*(OCNC)
812	0.8	779	0.3	802	s323-10 s327-30	*out*(OCNC)
		862	4.6	894		
				892	s108-52	ν(NC)
		761	2.2	783	s324-33	*out*(OCNC)
		750	7.0	774	s323-24	*out*(OCNC)
				772	s323-12	*out*(OCNC)
				771	s325-49	*out*(OCNC)
				770	s330-45	*out*(OCNC)
757	2.8			768	s326-52	*out*(OCNC)
				765	s232-13 s337-43	*out*(CCNC)+*out*(OCNC)
		775	0.7	798	s220-13 s328-42	β(CCN)+*out*(OCNC)
		738	0.7	758	s238-37	τ(HNCC)
		732	4.4	752	s267-35	*out*(CCCH)
		726	1.2	746	s169-11 s280-54	β(HCC)+ τ(HCCC)
		687	0.6	704	s181-26 s237-13	β(OCO)+ τ(HNCC)
		676	1.1	692	s237-31	τ(HNCC)
		694	1.8	712	s182-40	β(OCO)
683	3.2			710	s181-16 s237-10 s238-15	β(OCO)+ τ(HNCC)
		710	0.6	728	s265-11	τ(HCCC)
		720	1.1	739	s237-26 s238-16	τ(HNCC)
		659	0.8	674	s175-11	β(CCO)
		671	1.5	686		
661	3.9	652	0.8	666	s175-14	β(CCO)
		637	1.0	650	s178-10 s239-12	β(NCO)+ τ(HNCC)
		634	1.7	647	s176-12 s239-10	β(NCO)+ τ(HNCC)
		569	0.3	577	s251-11 s322-24 s329-16	τ(HCCO)+*out*(OCOC)+*out*(OCNC)
569	14.4	590	1.6	599	s190-12 s223-10 s233-16	β(CCN)+ τ(HNCC)
		593	0.9	602	s211-10 s239-14	β(CCN)+ τ(HNCC)

ν_{exp}	AR_{exp} %	ν_{scaled}	AR_{calc} %	ν_{calc}	PED%	Interpretation
		609	1.9	620	s233-59	τ(HNCC)
		604	0.6	615	s199-10 s239-45	β(NCO)+ τ(HNCC)
		557	2.3	565	s251-13 s322-25 s329-15	τ(HCCO)+*out*(OCOC)+*out*(OCNC)
				564	s230-37	τ(HNCC)
		548	1.0	556	s231-87	τ(HNCC)
557	14.2			553	s229-76	τ(HNCC)
		543	1.1	549	s228-14 s230-19	τ(HNCC)
		540	0.6	546	s228-30 s230-14	τ(HNCC)
		526	0.7	531	s228-40	τ(HNCC)
		505	1.1	508	s210-10 s218-17	β(CCN)
		430	1.3	427	s218-11	β(CCN)
		433	0.5	431	s202-34	β(CCN)
		407	2.1	403	s210-12	β(CCN)
		452	0.6	451		
		398	1.0	393		
427	1.6	390	1.6	384		
		464	1.3	464	s224-18	β(CCN)
		471	0.6	472	s333-10	*out*(CCNC)
		489	2.4	491	s191-56	β(CCN)
		499	2.7	502	s183-32 s336-11	β(CCO)+*out*(CCNC)
				499	s184-38	β(CCO)

Table S4. Interpretation of Raman SERS Spectrum of Oligopeptide $(AlaAsp)_2(AlaLys)_2$ (Ag_2/-C=O Terminal).

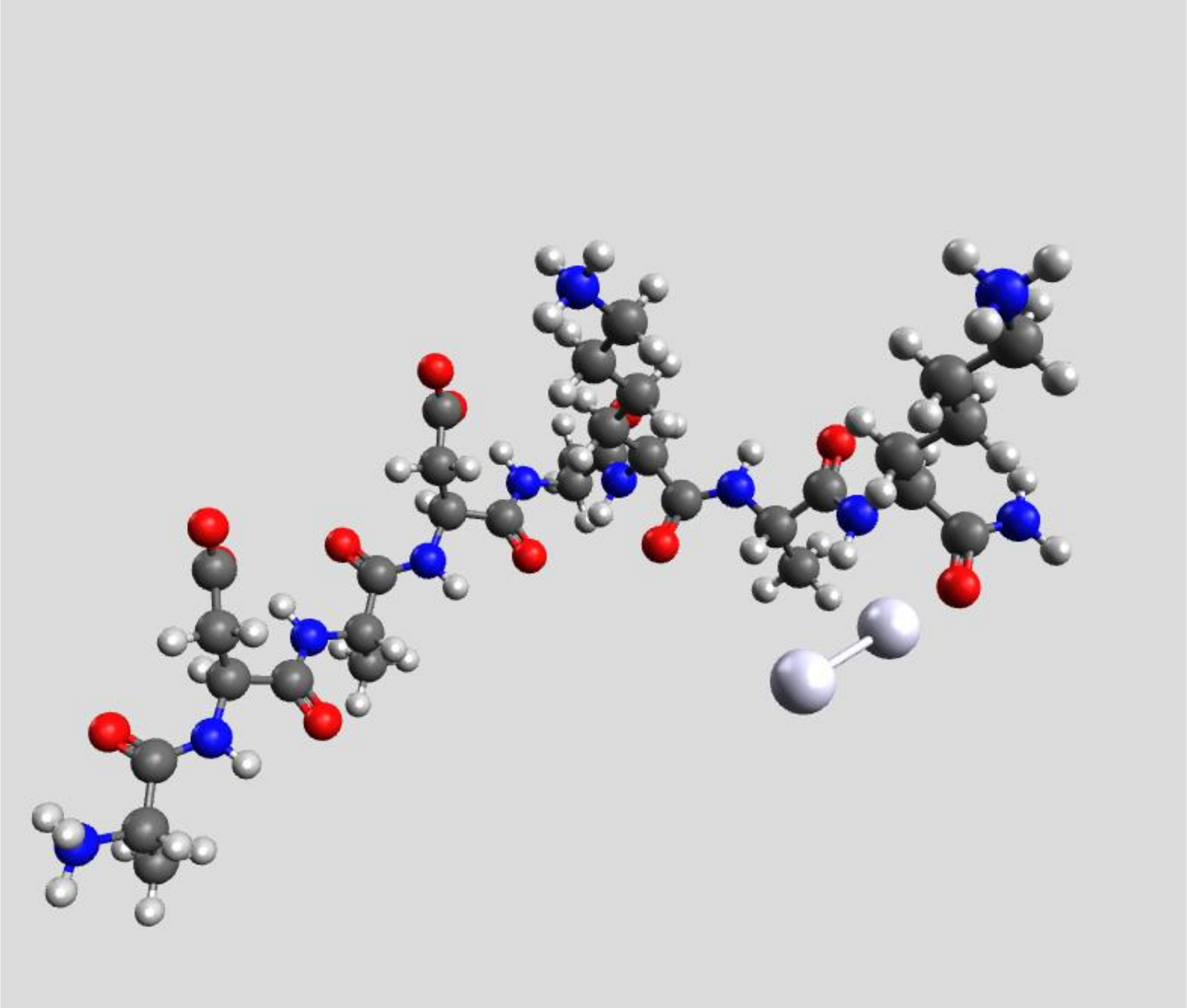

Table reports experimental frequencies (ν_{exp}, cm^{-1}), calculated frequencies (ν_{calc}, cm^{-1}), scaled frequencies ($\nu_{scaled} = 0.9313*\nu_{calc} + 29.80$; scaling straightline), percent experimental Raman Intensities (AR_{exp}), percent theoretical Raman Intensities (AR_{calc}), PED% (sxx-PED%, where sxx is the xx[th] internal coordinate) and mode Interpretation (ν = stretching, β = bending, τ = torsion, *out* = out-of-plane). Coalescence of several theoretical frequencies into a scaled frequency is the result of the PED fitting procedure.

ν_{exp}	AR_{exp} %	ν_{scaled}	AR_{calc}%	ν_{calc}	PED%	Interpretation
2972	29.8	2971	36.9	3160	s34-94	ν(CH)
				3159	s27-90	ν(CH)
				3158	s48-76 s49-17	ν(CH)
				3155	s29-89	ν(CH)
		2976	21.4	3163	s48-16 s49-81	ν(CH)
				3163	s35-94	ν(CH)

ν_{exp}	AR_{exp} %	ν_{scaled}	AR_{calc}%	ν_{calc}	PED%	Interpretation
		2965	15.2	3151	s19-83 s20-15	ν(CH)
		2981	8.8	3169	s19-12 s20-80	ν(CH)
		2987	6.5	3176	s22-88	ν(CH)
		2953	9.0	3138	s50-97	ν(CH)
		2990	6.4	3179	s44-98	ν(CH)
		2996	6.3	3185	s57-98	ν(CH)
		2933	16.2	3118	s58-99	ν(CH)
		2926	100.0	3113	s23-87 s24-11	ν(CH)
				3112	s45-99	ν(CH)
				3112	s26-95	ν(CH)
				3111	s40-83	ν(CH)
				3110	s30-92	ν(CH)
				3109	s46-96	ν(CH)
2932	100.0			3108	s53-89	ν(CH)
		2944	24.5	3130	s37-92	ν(CH)
				3129	s23-11 s24-89	ν(CH)
				3128	s31-92	ν(CH)
		2900	64.0	3083	s42-95	ν(CH)
				3081	s55-93	ν(CH)
		2917	2.0	3102	s38-87	ν(CH)
				3100	s51-88	ν(CH)
		2871	30.4	3051	s56-97	ν(CH)
				3049	s43-94	ν(CH)
		2863	42.9	3042	s41-95	ν(CH)
				3040	s52-90	ν(CH)
		2875	23.5	3055	s39-96	ν(CH)
		2858	99.7	3037	s54-90	ν(CH)
2868	26.8	2881	56.0	3062	s47-94	ν(CH)
				3060	s28-96	ν(CH)
		2888	52.5	3070	s21-97	ν(CH)
				3067	s36-98	ν(CH)
		2892	53.4	3074	s25-98	ν(CH)
				3073	s32-98	ν(CH)
2781	2.4	not explained				

ν_{exp}	AR_{exp} %	ν_{scaled}	AR_{calc}%	ν_{calc}	PED%	Interpretation
2742	3.2	out of range				
1677	21.7	1603	2.8	1689	s61-67	ν(OC)
				1689	s65-66	ν(OC)
1648	23.4	1634	22.5	1723	s64-64	ν(OC)
				1721	s62-67	ν(OC)
		1624	1.5	1712	s59-11 s60-60	ν(OC)
		1621	0.4	1708	s59-63	ν(OC)
1556	8.1	1552	1.6	1634	s132-76 s243-23	β(HNH)+ τ(HNCC)
		1566	4.4	1651	s131-84 s245-13	β(HNH)+*out*(NHCH)
				1649	s129-75 s240-20	β(HNH)+ τ(HNCC)
				1648	s121-81 s236-15	β(HNH)+*out*(NHCH)
		1581	1.5	1666	s128-77 s242-20	β(HNH)+ τ(HNCC)
		1594	3.8	1680	s63-75	ν(OC)
		1592	2.5	1677	s66-63	ν(OC)
1534	6.6	1534	3.6	1616	s72-11 s119-77	ν(NC)+β(HNH)
				1615	s73-51 s124-16	ν(NC)+β(HNC)
				1615	s122-71 s234-19	β(HNH)+ τ(HNCC)
		1538	2.7	1619	s68-32 s75-17 s125-32	ν(OC)+ ν(NC)+β(HNC)
1512	3.8	1510	2.8	1591	s67-26 s71-15 s115-17	ν(NC)+β(HNC)
				1588	s68-50 s125-12	ν(OC)+β(HNC)
		1516	6.4	1596	s78-25 s118-36	ν(NC)+β(HNC)
		1504	0.5	1583	s67-15 s71-14 s115-12 s124-10	ν(NC)+β(HNC)
		1500	1.8	1579	s76-23 s117-17 s126-13	ν(NC)+β(HNC)
1450	15.0	1450	0.2	1525	s130-97	β(HNH)
		1461	0.2	1536	s127-98	β(HNH)
		1431	0.7	1505	s123-91	β(HNH)
		1487	4.8	1567	s74-15 s116-12 s126-11	ν(NC)+β(HNC)
				1564	s74-12 s77-14 s126-17	ν(NC)+β(HNC)
1391	21.2	1385	32.4	1464	s134-76 s248-12	β(HCH)+*out*(CHCH)
				1463	s162-54	β(HCH)
				1460	s155-59	β(HCH)
				1459	s142-63	β(HCH)
				1458	s150-61	β(HCH)
				1458	s166-49 s168-14	β(HCH)

ν_{exp}	AR_{exp} %	ν_{scaled}	AR_{calc}%	ν_{calc}	PED%	Interpretation
				1456	s163-56 s276-11	β(HCH)+*out*(CHCH)
		1400	6.2	1474	s159-73	β(HCH)
				1471	s172-61	β(HCH)
				1469	s135-58 s246-13	β(HCH)+*out*(CCCH)
		1377	9.0	1447	s168-61	β(HCH)
		1371	15.9	1441	s69-11 s70-52	ν(OC)
				1441	s69-56 s251-13	ν(OC)+*out*(CCCH)
				1439	s158-11 s269-43	β(HCN)+τ(HCCN)
				1439	s172-16 s280-11 s284-32	β(HCH)+τ(HCCC)+τ(HCCN)
		1412	5.1	1485	s72-20 s97-11 s164-15	ν(NC)+ν(CC)+β(HCC)
		1416	0.7	1488	s170-56 s272-11	β(HCH)+τ(HCCH)
				1488	s157-59	β(HCH)
		1333	1.3	1399	s261-65	*out*(CHHH)
		1338	5.5	1405	s146-38	β(HCH)
				1404	s136-70	β(HCH)
		1328	1.1	1394	s161-65	β(HCH)
		1342	1.7	1409	s268-10	τ(HCCC)
		1346	4.8	1414	s69-14 s251-56	ν(OC)+*out*(CCCH)
1331	12.3			1414	s157-13 s281-13 s284-11	β(HCH)+τ(HCCC)+τ(HCCN)
		1316	4.4	1382	s141-84	β(HCH)
				1380	s147-10 s151-10 s161-22	β(HCC)+β(HCN)+β(HCH)
		1352	3.4	1421	s151-11 s162-13	β(HCN)+β(HCH)
				1419	s70-19	ν(OC)
		1320	0.3	1386	s138-11	β(HCC)
		1357	8.2	1425	s135-16 s246-24	β(HCH)+*out*(CCCH)
		1285	6.5	1350	s133-32 s250-27	β(HCC)+*out*(CCNH)
				1349	s257-52	*out*(CCNH)
				1348	s273-46	*out*(CCNH)
		1293	5.0	1356	s147-17	β(HCC)
		1277	18.1	1342	s169-47 s283-16	β(HCC)+τ(HCCN)
1288	11.9			1341	s250-11 s253-22	*out*(CCNH)+*out*(CCCH)
				1339	s260-29	*out*(CCNH)
				1338	s156-63	β(HCC)
		1300	9.2	1366	s264-20	*out*(CCNH)
				1365	s253-34	*out*(CCCH)
				1363	s171-66	β(HCN)
						β(HCN)+τ(HNCC)+τ(HCCH)

ν_{exp}	AR_{exp} %	ν_{scaled}	AR_{calc}%	ν_{calc}	PED%	Interpretation
				1361	s158-43 s242-11 s272-18	*out*(CCNH)
		1307	3.8	1372	s277-39	
		1261	6.2	1322	s118-10 s164-19 s273-11	β(HNC)+β(HCC)+*out*(CCNH)
		1266	1.7	1327	s125-10 s140-17 s260-24	β(HNC)+β(HCC)+*out*(CCNH)
1258	14.8	1253	0.5	1314	s152-43 s268-10	β(HCC)+ τ(HCCC)
		1269	2.9	1330	s151-20	β(HCN)
		1272	7.8	1334	s169-16 s279-16 s281-10	β(HCC)+ τ(HCCC)
		1231	8.5	1290	s139-22 s250-16	β(HCC)+*out*(CCNH)
		1249	2.0	1309	s164-17 s167-24	β(HCC)
		1228	0.7	1286	s146-10 s257-12 s259-13	β(HCH)+*out*(CCNH)+ τ(HCCO)
		1223	2.5	1281	s75-18 s125-12 s145-10 s259-12	ν(NC)+β(HNC)+β(HCC)+ τ(HCCO)
1238	13.1	1217	21.3	1277		
				1275	s282-11	τ(HCCC)
				1273	s124-20	β(HNC)
				1272	s118-10	β(HNC)
		1202	7.1	1258	s115-29 s138-23	β(HNC)+β(HCC)
		1179	1.7	1234	s117-15 s154-16	β(HNC)+β(HCC)
		1175	0.2	1229	s108-10 s234-23 s248-23	ν(CC)+ τ(HNCC)+*out*(CHCH)
		1147	9.9	1201	s80-14 s81-12	ν(NC)
1162	1.7			1199	s83-16	ν(NC)
		1185	10.9	1242	s165-21 s167-16 s285-12	β(HCC)+*out*(CCNH)
				1240	s74-15 s116-40 s140-10 s145-11	ν(NC)+β(HNC)+β(HCC)
		1193	5.1	1249	s117-12 s126-10 s154-22	β(HNC)+β(HCC)
		1206	2.2	1263	s77-12 s126-16 s160-10	ν(NC)+β(HNC)+β(HCC)
		1134	0.5	1186	s262-11	*out*(CHCH)
		1122	4.3	1173	s225-11	β(CCC)
1131	5.4	1141	7.0	1194	s137-63	β(HCC)
				1192	s144-59	β(HCC)
		1126	0.1	1177		
		1118	1.1	1169	s79-11 s86-10	ν(NC)+ ν(CC)
		1103	4.9	1152	s120-14	β(HNC)
		1112	2.6	1162	s79-11 s85-10 s89-10	ν(NC)+ ν(CC)
1102	12.4	1090	3.1	1140	s81-15 s89-12	ν(NC)+ ν(CC)
				1138	s84-11	ν(NC)
		1083	7.7	1132	s249-19	*out*(CHCH)

ν_{exp}	AR_{exp} %	ν_{scaled}	AR_{calc}%	ν_{calc}	PED%	Interpretation
				1131	s84-13	ν(NC)
		1077	1.1	1125	s79-11 s89-12 s236-12	ν(NC)+ ν(CC)+*out*(NHCH)
		1051	15.3	1097	s87-54	ν(CC)
				1095	s92-33 s120-10	ν(CC)+β(HNC)
		1045	1.4	1090	s101-20 s109-18	ν(CC)
		1039	15.8	1086	s101-15 s109-24	ν(CC)
1051	8.6			1084	s140-24 s256-20	β(HCC)+*out*(CHCH)
				1081	s160-15 s276-14	β(HCC)+*out*(CHCH)
		1064	4.8	1111	s106-33 s276-11	ν(CC)+*out*(CHCH)
				1110	s103-38 s255-10 s256-10	ν(CC)+*out*(CHCH)
		1068	4.6	1115	s82-10	ν(NC)
		1073	4.6	1120	s82-11 s107-10 s120-10	ν(NC)+ ν(CC)+β(HNC)
		1019	3.6	1063	s149-34	β(HCC)
		1027	6.3	1072	s95-47	ν(CC)
1018	8.6			1070	s92-12 s107-24	ν(CC)
		1033	5.7	1078	s88-61	ν(CC)
				1075	s105-57	ν(CC)
		974	2.6	1013	s167-17 s245-11	β(HCC)+*out*(NHCH)
		986	7.7	1027	s100-10 s145-16 s258-12	ν(CC)+β(HCC)+ τ(HCCO)
		982	1.9	1023	s154-23 s242-13	β(HCC)+ τ(HNCC)
974	12.6	967	1.4	1006	s89-21 s236-27 s249-31	ν(CC)+*out*(NHCH)+*out*(CHCH)
		990	4.3	1033	s91-62	ν(CC)
				1032	s98-57	ν(CC)
				1030	s99-11 s138-12 s252-15 s329-12	ν(CC)+β(HCC)+*out*(CCCH)+*out*(OC NC)
		957	1.1	996	s108-10 s234-34 s248-14	ν(CC)+ τ(HNCC)+*out*(CHCH)
		937	8.8	976	s255-13 s262-10	*out*(CHCH)
				974	s275-19	*out*(CHCH)
935	18.1	933	0.4	969		
		926	2.9	962		
		919	3.3	955	s111-22 s240-11	ν(CC)+ τ(HNCC)
		899	13.6	934	s102-54 s181-13	ν(CC)+β(OCO)
				933	s104-56 s184-11	ν(CC)+β(OCO)
900	22.3	895	2.7	929	s106-15	ν(CC)
		889	8.9	923	s176-14 s252-15	β(NCO)+*out*(CCCH)
		915	5.1	950	s112-50 s243-11 s245-12	ν(CC)+ τ(HNCC)+*out*(NHCH)

ν_{exp}	AR_{exp} %	ν_{scaled}	AR_{calc}%	ν_{calc}	PED%	Interpretation
				949	s111-18	ν(CC)
		878	13.4	910		
				910		
		866	5.0	898		
		862	8.4	894	s108-11	ν(CC)
				894	s108-38	ν(CC)
		811	0.9	838	s97-18 s285-13	ν(CC)+*out*(CCNH)
812	0.8	788	1.5	814	s152-11 s271-16	β(HCC)+*out*(CCNH)
		870	1.3	902	s103-22 s256-11	ν(CC)+*out*(CHCH)
		760	3.6	784	s325-37	*out*(OCNC)
		753	1.0	776	s322-41	*out*(OCNC)
		749	7.4	773	s326-45	*out*(OCNC)
				773	s278-10 s285-17 s331-26	τ(HCCC)+*out*(CCNH)+*out*(NCOC)
				770	s327-15 s330-31	*out*(OCNC)
757	2.8			768	s326-10 s327-18 s330-29	*out*(OCNC)
		739	2.2	762	s238-29 s325-10	τ(HNCC)+*out*(OCNC)
				760	s324-45	*out*(OCNC)
		775	1.8	801	s322-10 s327-10 s328-30	*out*(OCNC)
				799	s285-13 s331-31	*out*(CCNH)+*out*(NCOC)
		732	1.5	754	s156-12 s267-30	β(HCC)+ τ(HCCN)
		685	1.0	704	s237-10 s238-10	τ(HNCC)
		693	3.0	713	s181-10 s184-40	β(OCO)
683	3.2	690	0.6	709	s181-26 s184-11	β(OCO)
		707	2.3	727	s265-11	τ(HCCC)
		717	2.1	738	s238-17	τ(HNCC)
		722	2.4	743	s165-10 s169-14 s280-40	β(HCC)+ τ(HCCC)
		664	1.8	681	s173-16 s237-48	β(CCN)+ τ(HNCC)
		667	2.2	684		
		654	0.7	670		
661	3.9	634	3.7	649	s176-11 s199-11	β(NCO)+β(CCC)
		630	2.0	644	s176-12	β(NCO)
		616	5.3	630	s186-43	β(CCO)
		626	0.4	641	s233-66	τ(HNCC)
569	14.4	569	0.6	579	s297-13 s323-12 s329-17	*out*(OCOC)+ τ(HNCC)+*out*(OCNC)
		564	1.0	573	s323-75	τ(HNCC)

ν_{exp}	AR_{exp} %	ν_{scaled}	AR_{calc}%	ν_{calc}	PED%	Interpretation
		584	0.7	595	s239-59	τ(HNCC)
		590	0.8	602		
		594	1.6	606	s239-16	τ(HNCC)
		558	3.5	568	s297-15 s329-10	*out*(OCOC)+*out*(OCNC)
				567	s297-10	*out*(OCOC)
		545	0.7	554	s229-68	τ(HNCC)
557	14.2	527	7.1	533	s231-86	τ(HNCC)
		532	2.1	541	s229-11 s230-32	τ(HNCC)
				538	s230-25	τ(HNCC)
		504	1.6	509	s230-23 s333-14	τ(HNCC)+*out*(CCNC)
		429	6.8	431	s202-13	β(CCN)
				428	s202-27	β(CCN)
		409	3.7	407	s232-94	τ(HNCC)
		451	1.1	452	s336-10	*out*(CCNC)
		403	3.1	401	s175-13	β(CCO)
		463	0.9	465	s208-34	β(CCN)
427	1.6	383	1.0	380		
		386	5.6	383		
		469	0.5	472	s334-12	*out*(CCNC)
		486	2.6	490	s201-52	β(CCC)
		500	1.2	504	s183-25 s337-10	β(CCO)+*out*(CCNC)
		495	4.3	500	s182-34	β(CCO)

Table S5. Interpretation of Raman SERS Spectrum of Oligopeptide $(AlaGlu)_2(AlaOrn)_2$ (Ag_2/–COO^{-}_{Glu}).

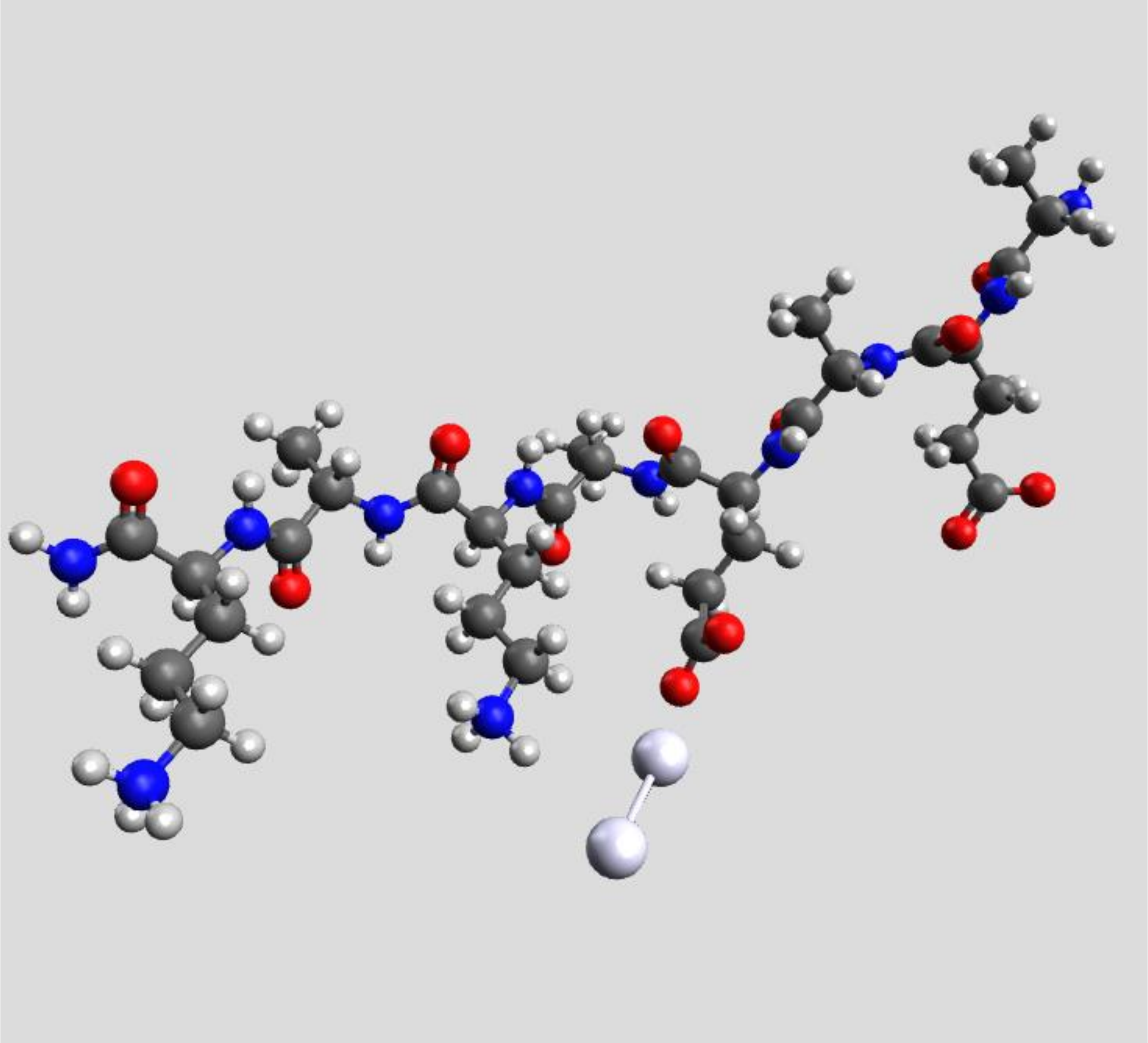

Table reports experimental frequencies (ν_{exp}, cm^{-1}), calculated frequencies (ν_{calc}, cm^{-1}), scaled frequencies ($\nu_{scaled} = 0.9284*\nu_{calc} + 36.94$; scaling straightline), percent experimental Raman Intensities (AR_{exp}), percent theoretical Raman Intensities (AR_{calc}), PED% (sxx-PED%, where sxx is the xxth internal coordinate) and mode Interpretation (ν = stretching, β = bending, τ = torsion, *out* = out-of-plane). Coalescence of several theoretical frequencies into a scaled frequency is the result of the PED fitting procedure.

ν_{exp}	AR_{exp} %	ν_{scaled}	AR_{calc}%	ν_{calc}	PED%	Interpretation
3071	1.6	3109	100.0	3309	s18-95	ν(NH)
		2993	2.9	3185	s57-98	ν(CH)
2973	31.3	2973	18.5	3164	s49-100	ν(CH)
				3163	s38-89	ν(CH)

ν_{exp}	AR_{exp} %	ν_{scaled}	AR_{calc}%	ν_{calc}	PED%	Interpretation
				3161	s29-97	ν(CH)
				3161	s40-87	ν(CH)
		2966	14.6	3157	s50-94	ν(CH)
				3155	s30-92	ν(CH)
				3153	s19-82 s20-16	ν(CH)
		2979	6.4	3170	s19-15 s20-83	ν(CH)
				3169	s46-96	ν(CH)
		2988	3.3	3179	s22-93	ν(CH)
		2930	27.1	3119	s28-95	ν(CH)
				3118	s52-10 s53-64 s58-21	ν(CH)
				3118	s48-95	ν(CH)
				3116	s53-19 s58-78	ν(CH)
				3115	s32-90	ν(CH)
				3114	s23-78 s24-19	ν(CH)
				3114	s42-14 s44-80	ν(CH)
		2941	13.0	3131	s52-85 s53-11	ν(CH)
2936	100.0			3129	s33-94	ν(CH)
				3128	s37-94	ν(CH)
				3127	s23-18 s24-75	ν(CH)
				3126	s41-96	ν(CH)
		2921	21.9	3107	s35-91	ν(CH)
				3105	s42-62 s44-14 s47-15	ν(CH)
		2917	33.2	3102	s42-11 s47-83	ν(CH)
				3102	s26-92	ν(CH)
				3102	s55-96	ν(CH)
		2873	46.0	3055	s45-91	ν(CH)
				3055	s54-13 s56-84	ν(CH)
				3054	s27-92	ν(CH)
		2881	79.7	3072	s21-96	ν(CH)
				3069	s34-96	ν(CH)
2874	29.1			3066	s39-97	ν(CH)
				3065	s25-94	ν(CH)
				3063	s43-81	ν(CH)
				3062	s51-97	ν(CH)
				3060	s31-97	ν(CH)
2786	1.5	not				

ν_{exp}	AR_{exp} %	ν_{scaled}	AR_{calc}%	ν_{calc}	PED%	Interpretation
		explai ned				
2737	2.3	out of range				
1676	19.0	not explai ned				
1638	20.7	1638	11.4	1726	s60-60	ν(OC)
				1723	s66-61	ν(OC)
		1630	0.4	1716	s61-60	ν(OC)
		1625	0.3	1711	s65-69	ν(OC)
1601	19.4	1602	4.1	1688	s59-69	ν(OC)
				1685	s64-74	ν(OC)
		1611	0.6	1695	s63-66	ν(OC)
		1618	0.6	1703	s62-73	ν(OC)
		1554	11.6	1634	s132-67 s240-21	β(HNH)+ τ(HNCC)
		1568	4.1	1650	s125-80 s239-14	β(HNH)+*out*(NHCH)
				1649	s130-72 s244-14	β(HNH)+*out*(NHCH)
1549	13.7			1649	s127-68 s241-19	β(HNH)+ τ(HNCC)
		1537	0.9	1616	s124-77 s237-15	β(HNH)+*out*(NHCH)
				1615	s74-11 s122-70 s123-12	ν(NC)+β(HNH)+β(HNC)
		1575	0.7	1657	s131-75 s243-14	β(HNH)+*out*(NHCH)
		1504	0.4	1580	s76-21 s121-25	ν(NC)+β(HNC)
		1508	1.0	1584	s71-10 s72-19 s116-23 s117-12	ν(NC)+β(HNC)
		1496	0.2	1572	s77-32 s118-46	ν(NC)+β(HNC)
		1514	0.7	1592	s73-21 s76-12 s120-22 s121-11	ν(NC)+β(HNC)
1502	7.3			1591	s68-91	ν(OC)
		1488	0.7	1563	s78-31 s119-28	ν(NC)+β(HNC)
		1523	1.5	1603	s67-12 s72-10 s75-13 s116-13 s117-21	ν(OC)+ ν(NC)+β(HNC)
				1600	s67-70	ν(OC)
		1518	0.3	1596	s71-18 s75-10 s115-22 s117-18	ν(NC)+β(HNC)
		1459	16.2	1532	s128-96	β(HNH)
				1532	s129-85	β(HNH)
1449	22.5	1445	0.3	1517	s126-91	β(HNH)
		1418	0.6	1488	s172-71	β(HCH)
				1487	s161-64 s273-24	β(HCH)+ τ(HCCH)

ν_{exp}	AR_{exp} %	ν_{scaled}	AR_{calc}%	ν_{calc}	PED%	Interpretation
		1396	5.5	1467	s170-63	β(HCH)
				1466	s157-64	β(HCH)
				1464	s139-46	β(HCH)
				1463	s134-72 s249-10	β(HCH)+*out*(CHCH)
				1462	s136-61 s256-11	β(HCH)+ τ(HCCN)
		1386	20.6	1459	s139-11 s148-43	β(HCH)
				1458	s164-47	β(HCH)
				1458	s143-46 s148-14 s257-12	β(HCH)+ τ(HCCN)
1394	37.3			1456	s163-54 s276-19	β(HCH)+ τ(HCCN)
				1454	s145-60	β(HCH)
				1454	s159-70	β(HCH)
				1451	s153-70 s266-12	β(HCH)+*out*(CHCH)
		1404	1.6	1473	s74-12 s99-10 s167-11	ν(NC)+ ν(CC)+ β(HCC)
				1473	s152-49 s246-16	β(HCH)+*out*(CCCH)
		1377	1.2	1444	s168-12 s284-38 s285-13	β(HCH)+ τ(HCCC)
				1443	s161-13 s274-49	β(HCH)+ τ(HCCC)
		1368	1.8	1433	s152-27 s246-17	β(HCH)+*out*(CCCH)
		1363	1.2	1428	s146-33	β(HCC)
		1321	2.3	1384	s151-59 s165-14	β(HCH)+ β(HCC)
				1382	s144-26 s250-11	β(HCH)+*out*(CCCH)
		1331	2.1	1398	s144-55	β(HCH)
				1397	s151-13 s268-10	β(HCH)+*out*(CCNH)
				1394	s154-77	β(HCH)
				1392	s165-27	β(HCC)
1324	18.0	1314	0.6	1376	s148-11 s260-39	β(HCH)+ τ(HCCC)
		1339	0.5	1402	s172-13 s279-15 s285-21	β(HCH)+ τ(HCCC)
				1402	s135-80	β(HCH)
		1358	8.7	1423	s262-50	*out*(CCCH)
				1423	s155-11 s262-21	β(HCC)+*out*(CCCH)
				1421	s69-25 s141-47	ν(OC)+ β(HCH)
		1351	0.5	1415	s246-18	*out*(CCCH)
		1303	4.9	1364	s171-40 s282-13	β(HCN)+ τ(HCCN)
		1310	3.0	1371	s160-28 s271-11	β(HCC)+ τ(HCCC)
1305	15.9			1371	s141-12 s252-23	β(HCH)+ τ(HCCC)
				1368	s171-19 s282-24	β(HCN)+ τ(HCCN)
		1295	2.0	1356	s166-26	β(HCC)

ν_{exp}	AR_{exp} %	ν_{scaled}	AR_{calc}%	ν_{calc}	PED%	Interpretation
				1355	s133-23 s255-10	*β*(HCC)+*out*(CCNH)
		1290	3.3	1351	s268-36	*out*(CCNH)
				1348	s166-19 s275-15	*β*(HCC)+*out*(CCNH)
		1285	0.8	1344	s255-55	*out*(CCNH)
		1265	4.2	1323	s147-32 s259-37	*β*(HCC)+*out*(CCNH)
		1252	2.3	1309	s74-10 s167-28	*ν*(NC)+*β*(HCC)
				1308	s138-61	*β*(HCC)
1261	13.5	1273	2.3	1331	s156-35 s264-10	*β*(HCC)+ *τ*(HCCN)
		1270	0.7	1328	s264-43	*τ*(HCCN)
		1277	2.4	1336	s140-11 s250-37	*β*(HCC)+*out*(CCCH)
				1335	s275-40	*out*(CCNH)
		1231	4.1	1286	s138-12	*β*(HCC)
		1240	0.3	1296	s269-65	*τ*(HCCC)
		1245	2.7	1303	s147-17 s150-24 s259-18	*β*(HCC)+*out*(CCNH)
				1300	s281-41	*τ*(HCCN)
1238	13.9	1223	2.5	1279	s115-10 s142-11	*β*(HNC)+*β*(HCC)
				1277	s120-18 s158-16 s272-11	*β*(HNC)+*β*(HCC)+ *τ*(HCCN)
		1216	1.2	1270	s77-20 s118-19 s146-10	*ν*(NC)+*β*(HNC)+*β*(HCC)
		1211	1.0	1265	s140-14 s149-12	*β*(HCC)
		1207	0.8	1260	s149-22	*β*(HCC)
		1153	4.9	1202	s83-12	*ν*(NC)
				1200	s80-23	*ν*(NC)
		1145	0.5	1194	s85-10	*ν*(NC)
		1189	4.0	1241	s119-11 s120-12 s121-11	*β*(HNC)
1159	7.7	1179	0.1	1230	s133-11 s237-23 s248-12 s249-12	*β*(HCC)+*out*(NHCH)+*out*(CHCH)
		1200	3.8	1256	s169-44	*β*(HCC)
				1254	s115-14 s253-17	*β*(HNC)+ *τ*(HCCO)
				1252	s158-18 s272-15	*β*(HCC)+ *τ*(HCCN)
		1193	0.5	1246	s78-11 s119-24 s121-11	*ν*(NC)+*β*(HNC)
		1128	4.0	1176		
				1175	s112-11 s244-19	*ν*(CC)+*out*(NHCH)
1126	17.4			1172	s137-48	*β*(HCC)
		1119	1.4	1166	s79-34	*ν*(NC)
		1137	2.0	1185	s241-13	*τ*(HNCC)
1104	18.2	1100	3.1	1145	s123-18 s283-15	*β*(HNC)+ *τ*(HCCN)

ν_{exp}	AR_{exp} %	ν_{scaled}	AR_{calc}%	ν_{calc}	PED%	Interpretation
		1107	0.4	1153	s90-11	ν(CC)
		1095	1.1	1140	s84-12	ν(NC)
		1092	1.0	1136	s107-10	ν(CC)
		1082	1.8	1125	s88-12	ν(CC)
		1086	4.5	1132	s88-10 s239-14	ν(CC)+*out*(NHCH)
1082	16.6			1130		
		1076	2.6	1120	s90-14	ν(CC)
		1072	2.7	1115	s102-21	ν(CC)
				1114	s103-21	ν(CC)
		1051	8.0	1095	s87-52	ν(CC)
				1093	s86-73	ν(CC)
				1090	s91-71	ν(CC)
		1043	9.9	1087	s81-34	ν(CC)
				1086	s162-15 s276-11	β(HCC)+τ(HCCN)
1051	13.9			1084	s81-23 s256-11	ν(CC)+τ(HCCN)
				1082	s110-25	ν(CC)
				1079	s97-23 s107-23	ν(NC)+ν(CC)
		1061	2.8	1103	s105-27	ν(CC)
		1067	1.1	1109	s74-10 s84-12 s123-21	ν(NC)+β(HNC)
		1030	1.5	1070	s100-47	ν(CC)
		1002	0.6	1040	s108-64 s244-10	ν(NC)+*out*(NHCH)
		1013	2.4	1052	s97-33	ν(NC)
1000	4.3			1050	s97-14 s261-17	ν(NC)+*out*(CCCH)
		1019	2.1	1058	s93-12 s140-18 s251-11 s254-13	ν(CC)+β(HCC)+τ(HCCC)+*out*(CCCH)
		978	0.8	1014	s88-35 s237-10 s239-14 s248-12	ν(CC)+*out*(NHCH)+*out*(CHCH)
		1027	1.6	1066	s106-11 s267-24	ν(CC)+*out*(CHCH)
		942	14.8	975	s111-22 s241-15	ν(CC)+τ(HNCC)
				973	s277-12	*out*(CHCH)
		934	5.1	969	s94-10 s256-16	ν(CC)+τ(HCCN)
951	18.1			967	s92-10 s248-17	ν(CC)+*out*(CHCH)
				964	s111-11 s112-13	ν(CC)
		966	1.8	1001	s237-26 s249-22	*out*(NHCH)+*out*(CHCH)
				1000	s243-20	*out*(NHCH)
		973	1.9	1009	s158-12 s240-19	β(HCC)+τ(HNCC)
908	16.7	908	11.6	939	s95-25	ν(CC)

ν_{exp}	AR_{exp} %	ν_{scaled}	AR_{calc}%	ν_{calc}	PED%	Interpretation
		915	2.7	946	s95-23	ν(CC)
		911	0.4	942		
		897	2.9	926	s101-16	ν(CC)
				926	s69-10 s101-26	ν(OC)+ ν(CC)
		924	1.3	956	s112-32 s244-10	ν(CC)+*out*(NHCH)
		873	0.2	901	s102-17 s257-11	ν(CC)+ τ(HCCN)
		864	3.3	890	s109-41 s173-10	ν(NC)+β(NCO)
873	3.8	889	3.8	918	s103-19	ν(CC)
		884	0.8	913	s177-15 s266-11	β(NCO)+*out*(CHCH)
		853	0.3	879	s285-10	τ(HCCC)
		836	0.6	860	s158-10 s272-13	β(HCC)+ τ(HCCN)
		765	0.6	786	s329-31	*out*(OCNC)
				784	s273-10 s329-18 s331-17	τ(HCCH)+*out*(OCNC)
		754	0.6	773	s328-50	*out*(OCNC)
		774	0.4	794	s194-10 s327-53	β(CCN)+*out*(OCNC)
		747	1.1	767	s323-43	*out*(OCNC)
				766	s322-62	*out*(OCNC)
				764	s325-57	*out*(OCNC)
763	3.9	781	0.6	801	s198-12 s330-52	β(CCN)+*out*(OCNC)
		742	0.9	760	s323-17 s331-27	*out*(OCNC)
		792	0.9	813	s99-22	ν(CC)
		721	2.8	738	s270-14	τ(HCCC)
				736	s251-10	τ(HCCC)
		736	0.2	753	s166-14 s280-37 s283-13 s329-10	β(HCC)+ τ(HCCH)+ τ(HCCN)+*out*(OCNC)
		800	0.6	822	s261-12 s263-24 s324-18	*out*(CCCH)+*out*(OCOC)
		806	0.3	829	s137-17 s286-16	β(HCC)+*out*(OCOC)
		671	0.3	683	s175-11	β(NCO)
		676	0.6	689	s181-11	β(OCO)
671	1.3	680	0.5	692	s182-55	β(OCO)
		691	2.8	704	s181-44	β(OCO)
		696	1.5	710		
		638	1.9	648	s174-42	β(NCO)
640	3.3	646	1.0	656	s178-41	β(NCO)
		630	0.4	639	s190-34 s236-13	β(CCO)+ τ(HNCC)
613	1.9	615	0.9	623	s236-20	τ(HNCC)

ν_{exp}	AR_{exp} %	ν_{scaled}	AR_{calc}%	ν_{calc}	PED%	Interpretation
				622	s236-39	τ(HNCC)
		599	3.7	605	s263-17 s324-32	*out*(CCCH)+*out*(OCOC)
		602	0.3	609	s236-12	τ(HNCC)
575	3.2	576	0.9	582	s229-70	τ(HNCC)
				580	s188-10 s229-11	β(CCN)+ τ(HNCC)
		582	1.0	587	s254-32 s286-44	*out*(CCCH)+*out*(OCOC)
		567	0.8	571	s232-23	τ(HNCC)
551	3.6	550	0.9	554	s232-52	τ(HNCC)
				552	s230-80	τ(HNCC)
		541	2.5	543	s234-74	τ(HNCC)
				543	s184-12 s231-29 s234-12	β(CCO)+ τ(HNCC)
		558	0.3	562	s228-81	τ(HNCC)
		562	0.2	565	s233-71	τ(HNCC)
516	2.8	518	1.7	518	s231-46	τ(HNCC)
		507	1.0	506	s205-32	β(CCN)
		503	1.2	502	s333-15	*out*(CCNC)
		497	1.1	496	s210-10	β(CCC)
466	3.2	462	1.7	458	s335-14	*out*(CCNC)
		455	0.8	450	s225-30	β(CCN)
		438	1.0	432	s225-17	β(CCN)
		433	1.0	427	s200-35	β(CCN)
362	2.4	366	2.3	354	s221-11	β(CCN)
		355	0.8	343	s173-13 s188-10	β(NCO)+β(CCN)
				342	s173-14 s222-10	β(NCO)+β(CCN)
		377	4.0	368	s235-96	τ(HNCC)
				367		
				365		
		346	0.7	333		
		338	3.2	324	s179-12 s245-13	β(CCO)+ τ(HNCC)
				323	s176-12	β(CCO)
		328	2.3	315	s332-17	*out*(CCNC)
				313	s242-64	τ(HNCC)
				311	s242-26	τ(HNCC)
		399	2.0	390	s214-19 s336-15	β(CCC)+*out*(CCNC)
				390		

ν_{exp}	AR_{exp} %	ν_{scaled}	AR_{calc}%	ν_{calc}	PED%	Interpretation
		334	17.5	320	s245-71	τ(HNCC)
		409	2.8	401	s210-34	β(CCC)
		421	0.7	414	s204-10 s213-15	β(CCC)

Table S6. Interpretation of Raman SERS Spectrum of Oligopeptide (AlaGlu)$_2$(AlaOrn)$_2$ (Ag$_2$/-C=O in the peptidic chain, 2nd Setting).

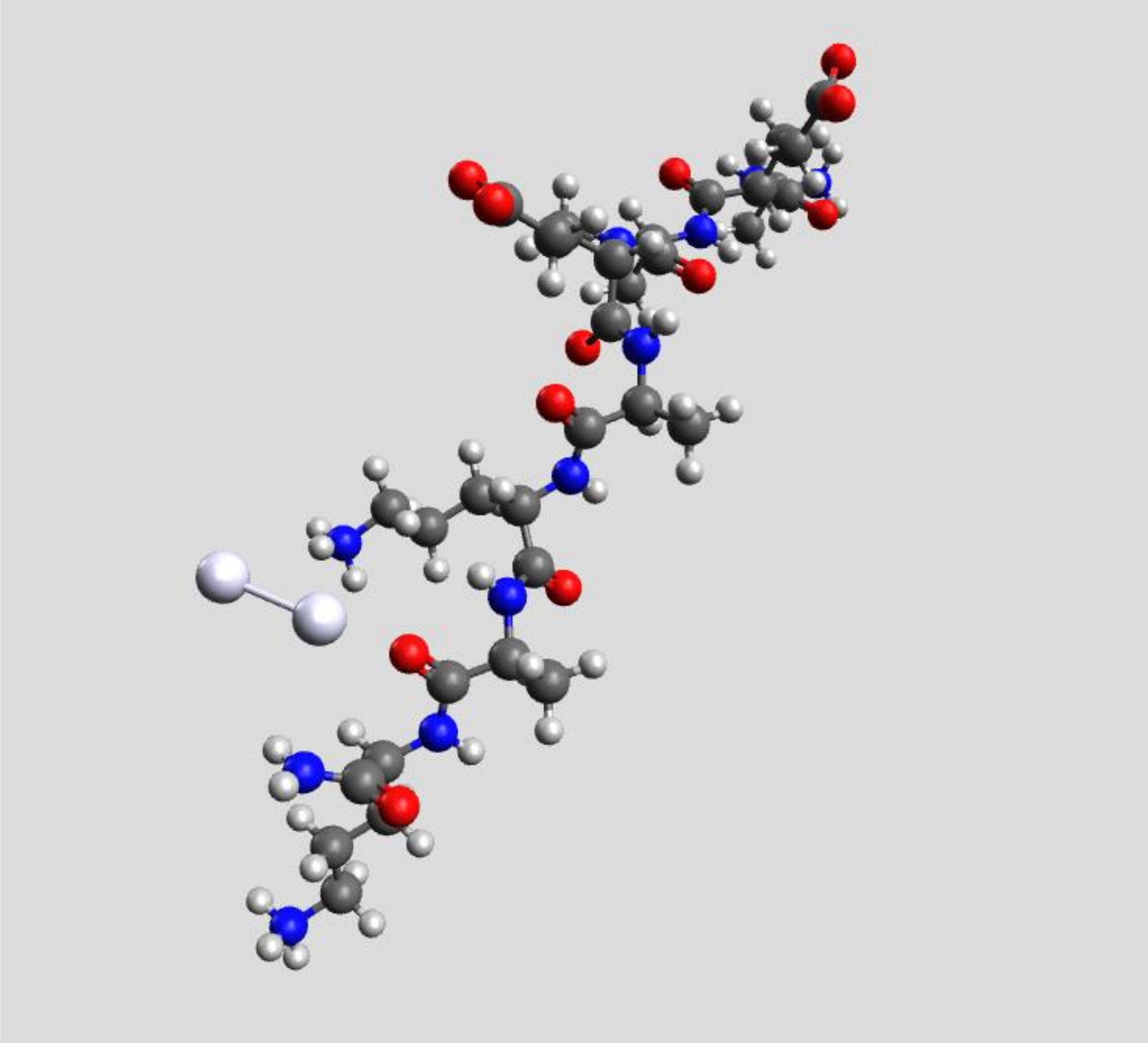

Table reports experimental frequencies (ν_{exp}, cm^{-1}), calculated frequencies (ν_{calc}, cm^{-1}), scaled frequencies (ν_{scaled} = 0.9348*ν_{calc} + 21.39; scaling straightline), percent experimental Raman Intensities (AR$_{exp}$), percent theoretical Raman Intensities (AR$_{calc}$), PED% (sxx-PED%, where sxx is the xxth internal coordinate) and mode Interpretation (ν = stretching, β = bending, τ = torsion, *out* = out-of-plane). Coalescence of several theoretical frequencies into a scaled frequency is the result of the PED fitting procedure.

ν_{exp}	AR$_{exp}$ %	ν_{scaled}	AR$_{calc}$%	ν_{calc}	PED%	Interpretation
3071	1.6	3000	2.2	3186	s57-98	ν(CH)
		3130	58.5	3325	s18-94	ν(NH)
2973	31.3	2973	13.4	3159	s29-97	ν(CH)

ν_{exp}	AR_{exp} %	ν_{scaled}	AR_{calc}%	ν_{calc}	PED%	Interpretation
				3159	s51-89	ν(CH)
				3156	s30-88	ν(CH)
				3155	s38-96	ν(CH)
		2978	4.2	3162	s49-98	ν(CH)
		2982	6.5	3167	s39-93	ν(CH)
				3167	s19-10 s20-87	ν(CH)
		2961	5.0	3145	s19-89	ν(CH)
		2993	6.3	3180	s21-24 s22-74	ν(CH)
				3178	s46-98	ν(CH)
		2953	2.1	3136	s41-89 s42-10	ν(CH)
		2936	10.1	3118	s23-80 s26-17	ν(CH)
				3118	s58-99	ν(CH)
				3118	s41-10 s42-74 s44-15	ν(CH)
		2931	7.2	3112	s47-99	ν(CH)
		2925	14.1	3106	s54-95	ν(CH)
2936	100.0			3105	s35-87	ν(CH)
		2948	10.5	3132	s48-91	ν(CH)
				3130	s28-90	ν(CH)
				3129	s25-90	ν(CH)
		2942	0.9	3124	s33-90	ν(CH)
				3123	s55-95	ν(CH)
		2920	7.1	3101	s23-14 s26-78	ν(CH)
		2871	10.7	3048	s45-97	ν(CH)
		2882	100.0	3070	s21-76 s22-23	ν(CH)
				3068	s24-97	ν(CH)
				3065	s34-90	ν(CH)
				3065	s53-83 s56-13	ν(CH)
2874	29.1			3063	s40-95	ν(CH)
				3062	s43-95	ν(CH)
				3061	s50-97	ν(CH)
		2902	5.5	3082	s37-99	ν(CH)
		2910	5.4	3090	s32-93	ν(CH)
		2915	7.3	3096	s42-15 s44-82	ν(CH)
2786	1.5	not explai				

ν_{exp}	AR_{exp} %	ν_{scaled}	AR_{calc}%	ν_{calc}	PED%	Interpretation
		ned				
2737	2.3	out of range				
1676	19.0	not explai ned				
1638	20.7	1634	7.6	1726	s63-62	ν(OC)
				1723	s61-63	ν(OC)
		1627	1.9	1717	s65-71	ν(OC)
		1624	1.0	1714	s59-64	ν(OC)
1601	19.4	1599	1.6	1688	s60-74	ν(OC)
		1608	0.7	1698	s62-64	ν(OC)
		1585	1.2	1673	s66-66	ν(OC)
		1615	0.7	1705	s64-68	ν(OC)
1549	13.7	1554	14.4	1639	s132-77 s245-21	β(HNH)+ τ(HNCC)
		1560	3.2	1647	s126-81 s239-14	β(HNH)+*out*(NHCH)
				1646	s127-74 s240-23	β(HNH)+ τ(HNCC)
				1644	s130-83 s243-13	β(HNH)+*out*(NHCH)
		1527	4.1	1612	s124-83 s237-13	β(HNH)+*out*(NHCH)
				1610	s76-13 s122-73 s123-10	ν(NC)+β(HNH)+β(HNC)
		1572	0.5	1658	s131-83 s244-12	β(HNH)+*out*(NHCH)
1502	7.3	1506	1.3	1590	s74-42 s115-10 s116-18	ν(OC)+β(HNC)
				1589	s71-85	ν(OC)
				1588	s67-70	ν(OC)
				1587	s77-10 s78-17 s119-14 s120-16 s121-11	ν(NC)+β(HNC)
		1495	1.5	1578	s69-18 s73-14 s117-36	ν(NC)+β(HNC)
				1576	s75-12 s77-13 s118-17 s119-15 s120-10	ν(NC)+β(HNC)
				1574	s75-20 s118-26 s119-13	ν(NC)+β(HNC)
		1516	0.3	1598	s72-28 s120-11 s121-28	ν(NC)+β(HNC)
				1598	s69-15 s73-15 s115-19 s116-16	ν(NC)+β(HNC)
1449	22.5	1454	7.2	1533	s128-98	β(HNH)
		1450	0.1	1528	s129-95	β(HNH)
		1426	0.1	1502	s125-93	β(HNH)
		1416	0.5	1492	s172-57 s284-10 s285-20	β(HCH)+ τ(HCCC)

ν_{exp}	AR_{exp} %	ν_{scaled}	AR_{calc}%	ν_{calc}	PED%	Interpretation
		1389	5.4	1468	s154-66	β(HCH)
				1466	s159-72	β(HCH)
				1463	s145-56	β(HCH)
				1463	s170-48	β(HCH)
				1462	s135-68 s248-10 s249-10	β(HCH)+*out*(CHCH)+τ(HCCN)
				1461	s148-62	β(HCH)
				1459	s165-60 s277-13	β(HCH)+*out*(CHCH)
		1399	0.9	1476	s168-63	β(HCH)
				1473	s134-44 s246-18 s249-10	β(HCH)+*out*(CCCH)+τ(HCCN)
1394	37.3	1379	12.1	1456	s153-72 s266-10 s267-10	β(HCH)+*out*(CHCH)
				1455	s139-68 s258-12	β(HCH)+*out*(CHCH)
				1454	s280-14 s281-17 s282-11 s283-14	*out*(CCCH)+τ(HCCN)
				1453	s143-68 s258-10	β(HCH)+*out*(CHCH)
				1453	s164-74	β(HCH)
				1451	s157-61 s278-10	β(HCH)+*out*(CHCH)
				1450	s70-36 s104-14 s149-11 s263-13	ν(OC)+ν(CC)+β(HCC)+τ(HCCO)
		1412	1.1	1487	s161-70 s273-11	β(HCH)+τ(HCCC)
		1366	1.1	1439	s112-10 s274-47	ν(CC)+τ(HCCC)
		1360	1.4	1432	s134-21 s246-22	β(HCH)+*out*(CCCH)
		1326	1.3	1396	s163-19	β(HCH)
				1394	s144-48 s250-12	β(HCH)+*out*(CCCH)
		1321	0.4	1390	s152-70	β(HCH)
		1318	0.7	1387	s163-63	β(HCH)
		1333	0.5	1403	s136-86	β(HCH)
1324	18.0			1403	s151-24	β(HCC)
		1339	1.5	1410	s167-26 s172-12 s279-11	β(HCC)+β(HCH)+*out*(CCNH)
		1350	5.5	1422	s151-12 s156-19	β(HCC)
				1422	s70-22 s150-49	ν(OC)+β(HCH)
				1420	s68-25 s141-45 s253-11	ν(OC)+β(HCH)+τ(HCCO)
		1345	0.5	1416	s142-13 s246-14	β(HCC)+*out*(CCCH)
		1356	0.6	1427	s146-39	β(HCC)
		1303	1.4	1371	s171-42 s284-13 s285-16	β(HCN)+τ(HCCC)
1305	15.9	1310	3.9	1379	s156-10 s279-12	β(HCC)+*out*(CCNH)
				1378	s150-13 s261-16	β(HCH)+τ(HCCC)

ν_{exp}	AR_{exp} %	ν_{scaled}	AR_{calc}%	ν_{calc}	PED%	Interpretation
		1300	1.4	1368	s141-12 s251-27	β(HCH)+ τ(HCCC)
		1314	1.0	1382	s144-36 s250-11 s251-10	β(HCH)+*out*(CCCH)+ τ(HCCC)
		1288	8.8	1357	s160-28 s271-16 s273-11	β(HCN)+ τ(HCCC)
				1356	s167-11 s279-13 s283-15	β(HCC)+*out*(CCNH)+ τ(HCCN)
				1355	s133-13 s255-22	β(HCC)+*out*(CCNH)
				1352	s160-24 s269-24 s274-16	β(HCN)+ τ(HCCN)+ τ(HCCC)
		1263	3.3	1330	s158-13 s275-44	β(HCC)+ τ(HCCN)
				1328	s147-17 s149-15 s259-27	β(HCC)+*out*(CCNH)
				1327	s76-11 s169-45	ν(NC)+β(HCC)
1261	13.5	1257	1.4	1321	s264-34	*out*(CCNH)
		1272	2.6	1339	s137-15 s250-35	β(HCC)+*out*(CCCH)
				1337	s158-25 s275-12	β(HCC)+ τ(HCCN)
		1277	0.4	1343	s133-14 s255-33	β(HCC)+*out*(CCNH)
		1281	0.9	1347	s166-27	β(HCC)
		1240	1.4	1303	s140-45	β(HCC)
		1232	1.2	1296	s268-46	τ(HCCC)
		1244	1.0	1308	s281-19 s282-13	τ(HCCN)
				1307	s140-16 s259-10	β(HCC)+*out*(CCNH)
		1223	2.8	1287		
1238	13.9			1285		
		1216	2.2	1278	s115-15	β(HNC)
		1213	2.0	1275	s162-11	β(HCC)
				1274	s118-10	β(HNC)
		1203	1.7	1265	s166-16 s167-11 s279-13	β(HCC)+*out*(CCNH)
				1264	s116-11 s137-10	β(HNC)+β(HCC)
		1159	1.5	1216	s79-22 s266-11	ν(NC)+*out*(CHCH)
		1143	1.9	1201	s81-11 s85-10	ν(NC)
				1200	s83-20	ν(NC)
		1171	0.1	1230	s133-10 s237-21 s248-24	β(HCC)+*out*(NHCH)+*out*(CHCH)
1159	7.7	1191	4.6	1252	s115-15 s117-11 s253-10	β(HNC)+ τ(HCCO)
				1250	s155-27	β(HCC)
		1195	1.7	1257	s117-14 s251-10 s253-10	β(HNC)+ τ(HCCC)+ τ(HCCO)
				1256	s77-17 s119-28	ν(NC)+β(HNC)
1126	17.4	1126	0.3	1182	s147-10 s261-15	β(HCC)+ τ(HCCC)

ν_{exp}	AR_{exp} %	ν_{scaled}	AR_{calc}%	ν_{calc}	PED%	Interpretation
		1132	2.3	1188		
		1118	1.8	1174		
				1172	s138-50	*β*(HCC)
		1102	0.9	1156	s106-15 s167-16	*ν*(CC)+*β*(HCC)
1104	18.2	1107	0.9	1161	s87-30	*ν*(CC)
		1095	0.4	1148	s89-14	*ν*(NC)
		1080	2.2	1134	s80-15	*ν*(NC)
				1131	s239-12 s249-15	*out*(NHCH)+*τ*(HCCN)
1082	16.6	1076	3.5	1128	s94-19	*ν*(CC)
		1086	0.5	1139	s270-25	*τ*(HCCN)
		1071	1.6	1124	s100-16	*ν*(CC)
				1122	s80-10	*ν*(NC)
		1055	1.7	1106	s103-28 s262-11	*ν*(CC)+*out*(CCCH)
		1042	7.4	1095	s88-48	*ν*(CC)
				1093	s86-50	*ν*(CC)
				1092	s112-61	*ν*(CC)
				1091	s84-38	*ν*(CC)
				1089	s123-19	*β*(HNC)
1051	13.9	1066	4.4	1119	s76-15 s106-13 s123-25	*ν*(NC)+*ν*(CC)+*β*(HNC)
				1117	s101-19 s278-10	*ν*(CC)+*out*(CHCH)
				1116	s109-31	*ν*(CC)
		1030	4.8	1080	s110-26	*ν*(CC)
				1080	s277-10	*out*(CHCH)
				1078	s95-37 s96-13	*ν*(CC)+*ν*(NC)
		1034	1.1	1084	s84-19 s257-11	*ν*(CC)+*out*(CHCH)
		1000	2.5	1048	s260-15 s262-10	*τ*(HCCC)+*out*(CCCH)
				1046	s96-57	*ν*(NC)
1000	4.3			1043	s98-63	*ν*(NC)
		1009	1.4	1056	s92-13 s137-21 s254-12	*ν*(CC)+*β*(HCC)+*out*(CCCH)
		1012	0.8	1060	s267-28	*out*(CHCH)
		951	0.4	995	s237-39 s248-13	*out*(NHCH)+*out*(CHCH)
951	18.1	962	8.9	1007	s158-10 s245-12	*β*(HCC)+*τ*(HNCC)
				1005	s87-22 s239-25 s249-26	*ν*(CC)+*out*(NHCH)+*τ*(HCCN)
		946	0.4	989	s171-11 s243-28 s285-10	*β*(HCN)+*out*(NHCH)+*τ*(HCCC)

ν_{exp}	AR_{exp} %	ν_{scaled}	AR_{calc}%	ν_{calc}	PED%	Interpretation
908	16.7	930	0.9	971	s105-10 s266-19	ν(CC)+*out*(CHCH)
		913	3.0	954	s90-23 s111-12 s244-14	ν(CC)+*out*(NHCH)
				954	s90-19 s111-11 s244-18	ν(CC)+*out*(NHCH)
		897	6.6	938		
				937		
		902	0.4	942		
		925	5.3	967	s91-14 s248-19	ν(CC)+*out*(CHCH)
				967	s257-20	*out*(CHCH)
				966	s111-11 s277-12	ν(CC)+*out*(CHCH)
873	3.8	872	1.0	909	s278-11	*out*(CHCH)
		868	0.9	905	s101-14	ν(CC)
		863	1.4	900	s109-10	ν(CC)
		885	2.3	925	s68-16 s99-42 s182-12	ν(OC)+ ν(CC)+β(OCO)
				924	s70-12 s104-32	ν(OC)+ ν(CC)
		852	3.5	889	s82-48	ν(NC)
				886	s108-12 s243-15 s281-14 s284-10	ν(CC)+*out*(NHCH)+ τ(HCCN)+ τ(HCCC)
		828	2.1	862	s272-15 s273-13	τ(HCCN)+ τ(HCCC)
763	3.9	760	1.8	790	s328-37	*out*(OCNC)
		766	0.4	796	s197-14 s327-44	β(CCN)+*out*(OCNC)
		756	0.5	786	s330-38	*out*(OCNC)
		778	1.7	809	s108-24	ν(CC)
		744	1.2	774	s329-52	*out*(OCNC)
				772	s337-25	*out*(OCNC)
		736	1.7	769	s325-38	*out*(OCNC)
				767	s323-22 s337-19	*out*(OCNC)
				764	s323-30 s325-11	*out*(OCNC)
				763	s326-56	*out*(OCNC)
		727	1.7	755	s108-11 s280-18 s282-20	ν(CC)+*out*(CCCH)+ τ(HCCN)
		795	0.3	829	s147-15 s263-15 s322-18	β(HCC)+ τ(HCCO)+*out*(OCOC)
				827	s252-27 s286-16	*out*(CCCH)+*out*(OCOC)
671	1.3	665	1.2	691	s182-32	β(OCO)
				688	s183-47	β(OCO)
		683	0.5	708	s176-15 s260-10 s334-10	β(CCO)+ τ(HCCC)+*out*(CCNC)
		660	0.2	683	s175-10 s182-18 s222-12	β(CCO)+β(OCO)+β(CCN)

ν_{exp}	AR_{exp} %	ν_{scaled}	AR_{calc}%	ν_{calc}	PED%	Interpretation
		690	0.2	716	s177-13	β(CCO)
		707	1.9	733	s272-20	τ(HCCN)
		711	1.2	738	s252-11 s272-11	*out*(CCCH)+ τ(HCCN)
640	3.3	641	0.6	663	s178-21	β(NCO)
		652	1.3	675	s179-25 s338-14	β(CCO)+*out*(CCNC)
613	1.9	604	1.6	624	s236-10	τ(HNCC)
		627	1.2	648	s174-47	β(NCO)
		598	7.9	617	s236-49	τ(HNCC)
575	3.2	573	0.8	590	s262-28 s322-48	*out*(CCCH)+*out*(OCOC)
		584	2.3	602	s236-14	τ(HNCC)
		562	0.4	578	s229-26	τ(HNCC)
551	3.6	559	0.5	575	s229-54	τ(HNCC)
		540	0.5	555	s190-12	β(CCN)
		569	0.6	585	s254-37 s286-48	*out*(CCCH)+*out*(OCOC)
		534	0.4	548	s233-67	τ(HNCC)
516	2.8	517	0.3	530	s232-80	τ(HNCC)
		514	0.4	527	s184-18 s335-11	β(CCO)+ τ(HNCC)
		522	0.3	536	s230-84	τ(HNCC)
		526	0.3	540	s228-78	τ(HNCC)
		500	0.3	512	s234-45	τ(HNCC)
		493	0.2	505	s181-12 s231-11	β(CCO)+ τ(HNCC)
466	3.2	482	2.0	493	s209-14 s332-15	β(CCN)+*out*(CCNC)
		486	0.5	497	s231-39 s335-20	τ(HNCC)
		490	1.0	501	s332-14	*out*(CCNC)
		437	1.3	444	s225-37	β(CCN)
		432	5.0	440	s211-14 s225-16	β(CCC)+β(CCN)
		445	0.2	453	s335-10	τ(HNCC)
		414	1.4	420	s331-12	*out*(CCNC)
362	2.4	360	3.2	362		
		370	0.5	373	s176-12 s192-10	β(CCO)+β(CCN)
		343	7.4	344	s180-11 s210-12 s235-16	β(NCO)+β(CCN)+ τ(HNCC)
		349	35.3	351	s235-16	τ(HNCC)
				350	s235-52	τ(HNCC)

ν_{exp}	AR_{exp} %	ν_{scaled}	AR_{calc}%	ν_{calc}	PED%	Interpretation
		385	1.3	389	s181-19 s336-15	*β*(CCO)+*out*(CCNC)
		339	0.7	339	s173-36	*β*(CCN)
		391	1.1	396	s184-12	*β*(CCO)
		327	1.9	327	s177-12 s207-10	*β*(CCO)+*β*(CCC)
		331	0.5	331	s334-12	*out*(CCNC)
		335	23.6	335	s241-88	*τ*(HNCC)
		320	1.2	320	s179-12 s338-10	*β*(CCO)+*out*(CCNC)
				317	s206-17 s238-15	*β*(CCN)+ *τ*(HNCC)
		399	0.4	404	s209-30 s332-11	*β*(CCN)+*out*(CCNC)
		408	0.4	413	s331-23	*out*(CCNC)

TableS7. Interpretation of Raman SERS Spectrum of Oligopeptide $(AlaGlu)_2(AlaOrn)_2$ (Ag_2/-C=O in the peptidic chain, 1st setting).

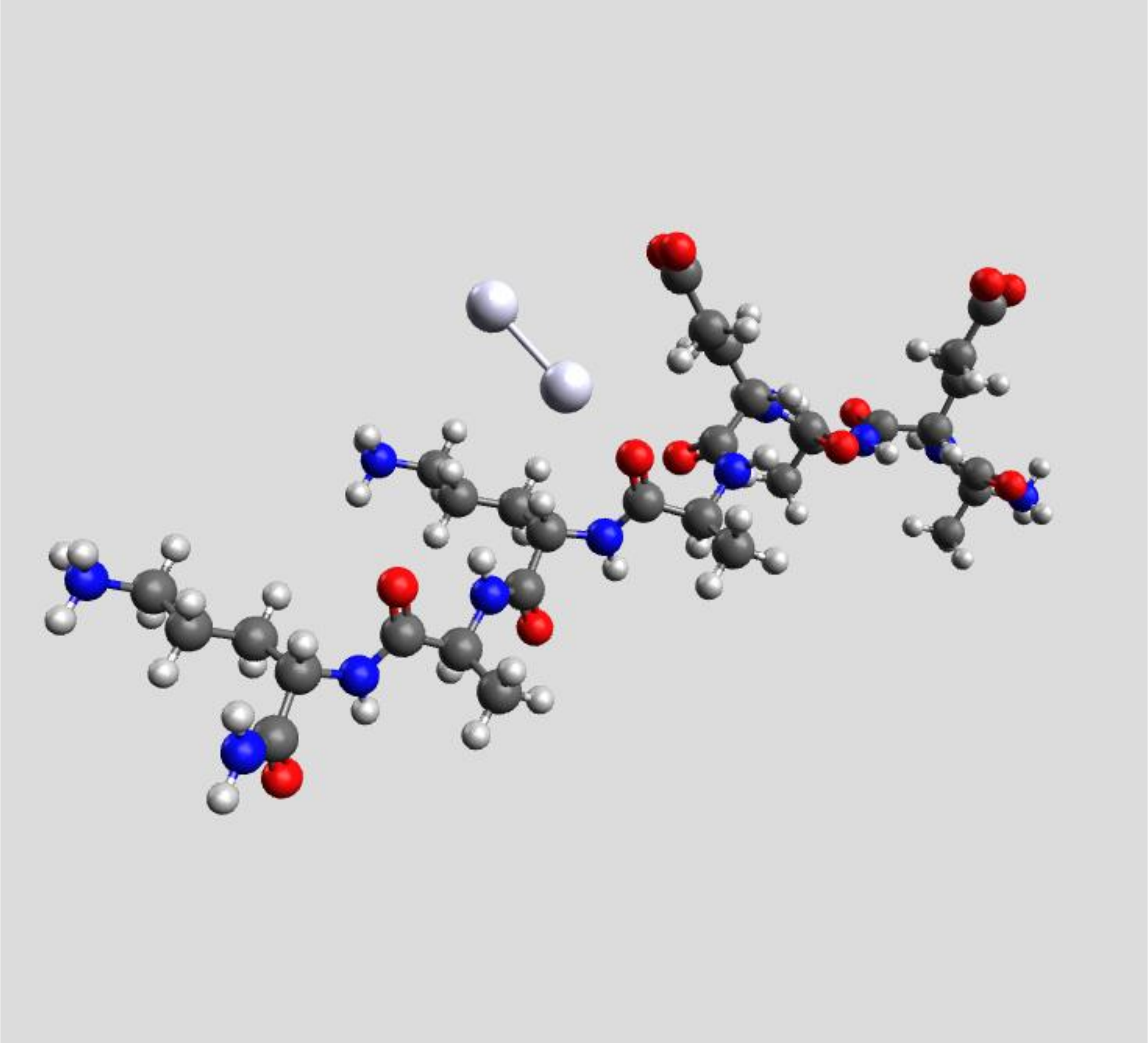

Table reports experimental frequencies (ν_{exp}, cm^{-1}), calculated frequencies (ν_{calc}, cm^{-1}), scaled frequencies ($\nu_{scaled} = 0.9316*\nu_{calc} + 33.01$; scaling straightline), percent experimental Raman Intensities (AR_{exp}), percent theoretical Raman Intensities (AR_{calc}), PED% (sxx-PED%, where sxx is the xxth internal coordinate) and mode Interpretation (ν = stretching, β□= bending, τ = torsion, *out* = out-of-plane). Coalescence of several theoretical frequencies into a scaled frequency is the result of the PED fitting procedure.

ν_{exp}	AR_{exp} %	ν_{scaled}	AR_{calc}%	ν_{calc}	PED%	Interpretation
3071	1.6	not explai ned				
2973	31.3	2973	25.6	3159	s38-98	ν(CH)

ν_{exp}	AR_{exp} %	ν_{scaled}	AR_{calc}%	ν_{calc}	PED%	Interpretation
				3157	s50-94	ν(CH)
				3155	s30-91	ν(CH)
				3153	s19-82 s20-17	ν(CH)
		2979	6.3	3162	s29-95	ν(CH)
		2986	10.8	3170	s19-14 s20-80	ν(CH)
				3170	s39-96	ν(CH)
		2981	6.4	3165	s49-98	ν(CH)
		2996	11.4	3183	s57-98	ν(CH)
				3180	s46-88	ν(CH)
				3180	s22-81 s46-10	ν(CH)
		2950	3.9	3131	s52-75 s53-22	ν(CH)
		2933	37.5	3116	s23-75 s25-14	ν(CH)
				3115	s58-99	ν(CH)
				3114	s32-95	ν(CH)
				3112	s47-96	ν(CH)
				3110	s28-96	ν(CH)
				3110	s41-91	ν(CH)
		2920	40.2	3100	s34-12 s35-83	ν(CH)
2936	100.0			3099	s23-15 s26-79	ν(CH)
				3096	s44-84	ν(CH)
		2926	22.7	3106	s55-96	ν(CH)
				3105	s48-97	ν(CH)
		2940	7.2	3120	s52-21 s53-76	ν(CH)
				3120	s42-89	ν(CH)
		2947	4.1	3128	s25-82	ν(CH)
				3127	s34-86 s35-13	ν(CH)
		2873	43.0	3048	s45-89	ν(CH)
		2878	47.1	3054	s36-96	ν(CH)
				3054	s27-97	ν(CH)
				3053	s43-88	ν(CH)
2874	29.1	2886	100.0	3068	s24-97	ν(CH)
				3067	s33-98	ν(CH)
				3066	s40-96	ν(CH)
				3063	s54-96	ν(CH)
				3062	s51-97	ν(CH)
				3060	s31-98	ν(CH)

ν_{exp}	AR_{exp} %	ν_{scaled}	AR_{calc}%	ν_{calc}	PED%	Interpretation
				3059	s56-96	ν(CH)
		2895	15.8	3072	s21-96	ν(CH)
		2911	10.4	3089	s37-99	ν(CH)
2786	1.5	not explained				
2737	2.3	out of range				
1676	19.0	not explained				
		1639	14.1	1725	s60-68	ν(OC)
				1722	s65-68	ν(OC)
1638	20.7	1631	0.6	1715	s61-60	ν(OC)
		1619	0.8	1703	s62-65	ν(OC)
		1625	0.4	1709	s63-64	ν(OC)
		1606	4.1	1690	s64-69	ν(OC)
				1687	s59-66	ν(OC)
1601	19.4	1593	3.0	1674	s66-59	ν(OC)
		1586	0.7	1667	s125-84 s239-12	β(HNH)+*out*(NHCH)
		1567	1.9	1648	s129-84 s241-14	β(HNH)+*out*(NHCH)
				1646	s132-81 s245-11	β(HNH)+*out*(NHCH)
1549	13.7	1541	0.8	1618	s196-80 s237-15	β(HNH)+*out*(NHCH)
		1575	1.9	1657	s131-72 s243-22	β(HNH)+ τ(HNCC)
				1654	s127-77 s240-21	β(HNH)+ τ(HNCC)
		1495	3.2	1569	s72-24 s116-19	ν(NC)+β(HNC)
				1569	s74-18 s78-15 s120-15 s121-16	ν(NC)+β(HNC)
		1513	2.4	1591	s72-12 s76-13 s116-12 s117-16	ν(NC)+β(HNC)
				1590	s67-91	ν(OC)
				1588	s69-82	ν(OC)
1502	7.3			1586	s73-14 s78-16 s119-13 s121-15	ν(NC)+β(HNC)
				1585	s71-18 s76-14 s115-17 s117-18	ν(NC)+β(HNC)
		1498	0.3	1573	s77-34 s118-43	ν(NC)+β(HNC)
		1529	0.4	1606	s122-76	β(HNH)
		1519	0.2	1595	s73-18 s74-12 s119-22 s120-12	ν(NC)+β(HNC)
1449	22.5	1447	0.3	1517	s126-91	β(HNH)

ν_{exp}	AR_{exp} %	ν_{scaled}	AR_{calc}%	ν_{calc}	PED%	Interpretation
		1417	2.8	1485	s159-71	β(HCH)
		1457	0.2	1529	s130-93	β(HNH)
				1528	s128-97	β(HNH)
		1424	0.4	1493	s172-59 s279-20	β(HCH)+ τ(HCCH)
		1393	18.3	1473	s170-31	β(HCH)
				1470	s136-53 s247-11	β(HCH)+ τ(HCCN)
				1467	s153-43 s265-11	β(HCH)+ τ(HCCN)
				1465	s161-60	β(HCH)
				1464	s153-13 s164-10 s170-20	β(HCH)
				1463	s134-54 s248-11	β(HCH)+ τ(HCCN)
1394	37.3			1462	s148-64 s247-10	β(HCH)+ τ(HCCN)
		1383	20.5	1449	s157-49	β(HCH)
				1449	s165-11 s168-37	β(HCH)
				1449	s70-40 s104-12 s263-12	ν(OC)+ ν(CC)+ τ(HCCO)
				1448	s68-40 s100-13 s254-13	ν(OC)+ ν(CC)+ τ(HCCO)
		1377	5.6	1442	s274-11 s281-11 s282-16	τ(HCCN)+ τ(HCCC)
				1442	s274-35	τ(HCCN)
		1330	2.7	1395	s154-19	β(HCC)
				1392	s163-83	β(HCH)
				1392	s143-63	β(HCH)
				1389	s154-57	β(HCC)
		1320	2.0	1381	s250-14 s252-17	*out*(CCCH)+ τ(HCCC)
		1356	10.8	1423	s151-14 s155-17	β(HCC)
1324	18.0			1422	s146-11 s150-10	β(HCC)+β(HCH)
				1420	s68-16 s141-31 s150-19	ν(OC)+β(HCH)
				1419	s141-17 s150-21	β(HCH)
		1336	0.9	1401	s135-24 s151-20	β(HCH)+β(HCC)
				1400	s135-33 s151-10 s246-13	β(HCH)+β(HCC)+*out*(CCCH)
				1398	s172-16 s279-14 s281-14	β(HCH)+ τ(HCCH)+ τ(HCCC)
		1344	0.5	1407	s135-27 s246-10	β(HCH)+*out*(CCCH)
		1363	1.4	1427	s134-11 s146-10	β(HCH)+β(HCC)
		1305	6.1	1368	s252-15	τ(HCCC)
				1365	s171-43 s279-14	β(HCN)+ τ(HCCH)
1305	15.9			1363	s160-35 s271-10 s273-12	β(HCN)+ τ(HCCC)
		1296	5.6	1356	s268-31	*out*(CCNH)
				1354	s167-39 s268-11	β(HCC)+*out*(CCNH)

ν_{exp}	AR_{exp} %	ν_{scaled}	AR_{calc}%	ν_{calc}	PED%	Interpretation
		1313	2.1	1375	s150-17 s260-37	β(HCH)+ τ(HCCC)
				1373	s285-45	*out*(CCNH)
		1289	3.9	1348	s156-10 s167-13 s275-16	β(HCC)+*out*(CCNH)
		1292	1.7	1351	s133-13 s137-20 s250-10	β(HCC)+*out*(CCCH)
		1261	8.0	1320	s259-38	τ(HCCN)
				1318	s147-23 s149-10 s264-23	β(HCC)+ τ(HCCN)
		1251	3.5	1308	s140-53 s253-13 s254-13	β(HCC)+ τ(HCCO)
1261	13.5			1305	s281-28	τ(HCCC)
		1282	6.1	1341	s156-39	β(HCC)
				1340	s255-45	τ(HCCN)
				1337	s133-26 s250-24	β(HCC)+*out*(CCCH)
		1275	1.5	1333	s275-46	*out*(CCNH)
		1239	2.1	1294	s162-10 s166-15	β(HCC)
		1246	4.0	1302	s263-18	τ(HCCO)
				1300	s270-22	τ(HCCC)
		1225	3.5	1280	s142-10 s263-10	β(HCC)+ τ(HCCO)
1238	13.9			1278	s120-12 s158-12	β(HNC)+β(HCC)
		1205	4.1	1259	s73-12 s119-27	ν(NC)+β(HNC)
				1258	s116-10	β(HNC)
		1219	1.8	1273	s77-12 s79-10 s118-11	ν(NC)+β(HNC)
		1214	1.5	1267	s115-10 s254-15	β(HNC)+ τ(HCCO)
		1164	3.0	1214	s79-20 s265-13	ν(NC)+ τ(HCCN)
		1146	3.1	1196	s81-22	ν(NC)
				1194	s84-18	ν(NC)
		1184	4.7	1236	s115-10 s116-21	β(HNC)
1159	7.7	1200	5.8	1254	s169-20 s283-10	β(HCC)+ τ(HCCC)
				1251	s158-11 s166-14	β(HCC)
				1249	s115-19	β(HNC)
		1190	2.9	1242	s78-11 s120-13 s121-22 s162-15	ν(NC)+β(HNC)+β(HCC)
		1173	0.2	1223	s237-21 s247-13	*out*(NHCH)+ τ(HCCN)
		1124	3.4	1173	s138-37	β(HCC)
1126	17.4			1170	s245-11	*out*(NHCH)
		1132	1.2	1180		
		1129	0.6	1176	s147-13 s149-15	β(HCC)
1104	18.2	1094	2.4	1139	s283-25 s284-10	τ(HCCC)

ν_{exp}	AR_{exp} %	ν_{scaled}	AR_{calc}%	ν_{calc}	PED%	Interpretation
		1113	1.6	1159	s80-44	ν(NC)
		1097	1.1	1142	s85-10 s272-15	ν(NC)+ τ(HCCN)
		1100	0.2	1145	s90-12	ν(NC)
		1079	6.6	1123	s83-16	ν(NC)
				1122		
		1085	4.7	1130	s96-13	ν(CC)
1082	16.6			1128	s239-14	*out*(NHCH)
		1071	3.1	1115	s101-24	ν(CC)
				1114	s107-38 s278-16	ν(CC)+ τ(HCCN)
		1090	0.6	1135	s85-13	ν(NC)
		1048	9.6	1091	s87-61	ν(CC)
				1090	s86-66	ν(CC)
				1087	s82-52	ν(CC)
		1041	13.5	1084	s97-27	ν(CC)
1051	13.9			1083	s110-25	ν(CC)
				1081	s142-16	β(HCC)
				1079	s162-15 s276-25	β(HCC)+ τ(HCCN)
		1054	2.7	1097	s75-13 s123-33	ν(NC)+β(HNC)
				1096	s88-57	ν(CC)
		1061	2.9	1103	s103-34	ν(CC)
		1002	0.8	1040	s108-58	ν(NC)
		1010	2.1	1048	s102-11 s146-10 s261-18	ν(CC)+β(HCC)+*out*(CCCH)
1000	4.3	1006	1.2	1044	s91-70	ν(NC)
		1020	2.2	1059	s94-10 s137-18 s251-11	ν(CC)+β(HCC)+ τ(HCCC)
		1027	1.6	1067	s92-44	ν(CC)
		1024	1.1	1063	s105-10 s266-17	ν(CC)+ τ(HCCN)
		938	6.9	974	s265-17	τ(HCCN)
				971		
				970	s93-15 s247-21	ν(CC)+ τ(HCCN)
951	18.1	972	6.2	1009	s158-15 s241-17	β(HCC)+*out*(NHCH)
				1006	s239-26 s248-26	*out*(NHCH)+ τ(HCCN)
		932	3.3	965	s95-12 s257-25	ν(CC)+*out*(CHCH)
		959	0.9	994	s237-39	*out*(NHCH)
		967	1.8	1003	s169-12 s171-11 s243-21	β(HCC)+β(HCN)+ τ(HNCC)
908	16.7	905	10.1	936	s215-11	β(CCN)

ν_{exp}	AR_{exp} %	ν_{scaled}	AR_{calc}%	ν_{calc}	PED%	Interpretation
		893	9.0	926	s70-13 s104-31	ν(OC)+ ν(CC)
				925	s68-15 s100-40 s181-11	ν(OC)+ ν(CC)+β(OCO)
				922	s107-20 s278-10	ν(CC)+ τ(HCCN)
		918	3.0	950	s112-46 s245-24	ν(NC)+*out*(NHCH)
		913	1.6	946		
				943	s106-12 s111-18 s241-15	ν(CC)+*out*(NHCH)
		925	1.5	958	s111-25 s240-19	ν(CC)+ τ(HNCC)
		861	4.8	888	s109-46	ν(NC)
		870	0.6	898	s89-24 s258-13	ν(CC)+*out*(CHCH)
873	3.8	835	3.4	861	s273-12	τ(HCCC)
		882	0.4	912	s96-10	ν(CC)
		855	0.6	883		
		754	4.9	775	s324-34	*out*(OCNC)
				774		
		765	1.3	786	s330-48	*out*(OCNC)
				785	s328-46	*out*(OCNC)
		745	4.1	766	s329-52	*out*(OCNC)
				764	s323-57	*out*(OCNC)
				763	s337-47	*out*(OCNC)
763	3.9			762	s326-38 s337-11	*out*(OCNC)
		780	0.6	802	s195-10 s327-45	β(CCN)+*out*(OCNC)
		793	1.0	816	s99-21 s284-15	ν(CC)+ τ(HCCC)
		717	10.3	736		
				734		
		802	1.0	825	s261-18 s322-13	*out*(CCCH)+*out*(OCOC)
		736	0.3	755	s167-10 s280-47	β(HCC)+ τ(HCCH)
		806	0.3	829	s325-14	*out*(OCOC)
		669	0.5	682	s174-21 s181-10	β(NCO)+β(OCO)
		681	0.9	695	s183-43	β(OCO)
671	1.3	676	0.4	690	s181-54	β(OCO)
		687	1.4	702	s176-15	β(CCO)
		694	2.1	709	s180-14	β(CCO)
640	3.3	637	2.0	649	s175-49	β(NCO)
		650	1.8	663	s177-22 s197-13	β(NCO)+β(CCO)
613	1.9	614	0.5	623	s179-12 s236-19	β(CCN)+ τ(HNCC)

ν_{exp}	AR_{exp} %	ν_{scaled}	AR_{calc}%	ν_{calc}	PED%	Interpretation
		610	0.3	619	s236-40	τ(HNCC)
		622	0.6	632	s188-43	β(NCO)
		598	0.2	607	s179-13 s236-17	β(CCN)+ τ(HNCC)
		577	2.3	583	s232-34	τ(HNCC)
575	3.2	581	2.1	588	s232-11 s253-23 s325-29	τ(HNCC)+ τ(HCCO)+*out*(OCOC)
				588	s232-39 s253-10 s325-13	τ(HNCC)+ τ(HCCO)+*out*(OCOC)
		588	7.5	596	s262-12 s322-29	τ(HCCO)+*out*(OCOC)
		557	1.5	562		
551	3.6	545	0.5	549	s228-81	τ(HNCC)
		563	0.6	569	s229-79	τ(HNCC)
				567	s233-54	τ(HNCC)
		520	1.1	523	s230-29	τ(HNCC)
		500	1.8	502	s182-19 s201-10	β(CCO)+β(CCN)
516	2.8			499	s224-10	β(CCN)
		511	0.5	513	s332-11	*out*(CCNC)
		531	1.4	535	s234-83	τ(HNCC)
				533	s230-54	τ(HNCC)
		452	1.4	450		
		443	0.8	440	s187-11 s225-11	β(CCN)
466	3.2	429	1.2	425	s331-10	*out*(CCNC)
		423	1.2	419	s331-21	*out*(CCNC)
		489	0.4	489	s231-46	τ(HNCC)
		361	2.6	352	s235-93	τ(HNCC)
		364	3.0	355	s220-11 s221-12	β(CCC)+β(CCN)
				355	s173-36	β(CCN)
		350	1.8	340	s187-17	β(CCN)
		353	5.4	343	s209-17	β(CCC)
		379	3.1	372	s192-13	β(CCN)
362	2.4	369	0.4	360	s216-10	β(CCC)
		408	2.4	402	s204-13	β(CCC)
		396	1.2	389	s182-13 s336-12	β(CCO)+*out*(CCNC)
		357	0.1	348	s244-86	τ(HNCC)
		341	0.5	330		
		332	0.6	321	s177-12 s197-32	β(NCO)+β(CCO)
		415	0.9	410		

ν_{exp}	AR_{exp} %	ν_{scaled}	AR_{calc}%	ν_{calc}	PED%	Interpretation
		404	0.3	398		
		323	0.2	312	s176-11 s186-12 s195-13	β(CCO)+β(CNC)+β(CCN)

Table S8. Interpretation of Raman SERS Spectrum of Oligopeptide $(AlaGlu)_2(AlaOrn)_2$ (Ag_2/-C=O Terminal).

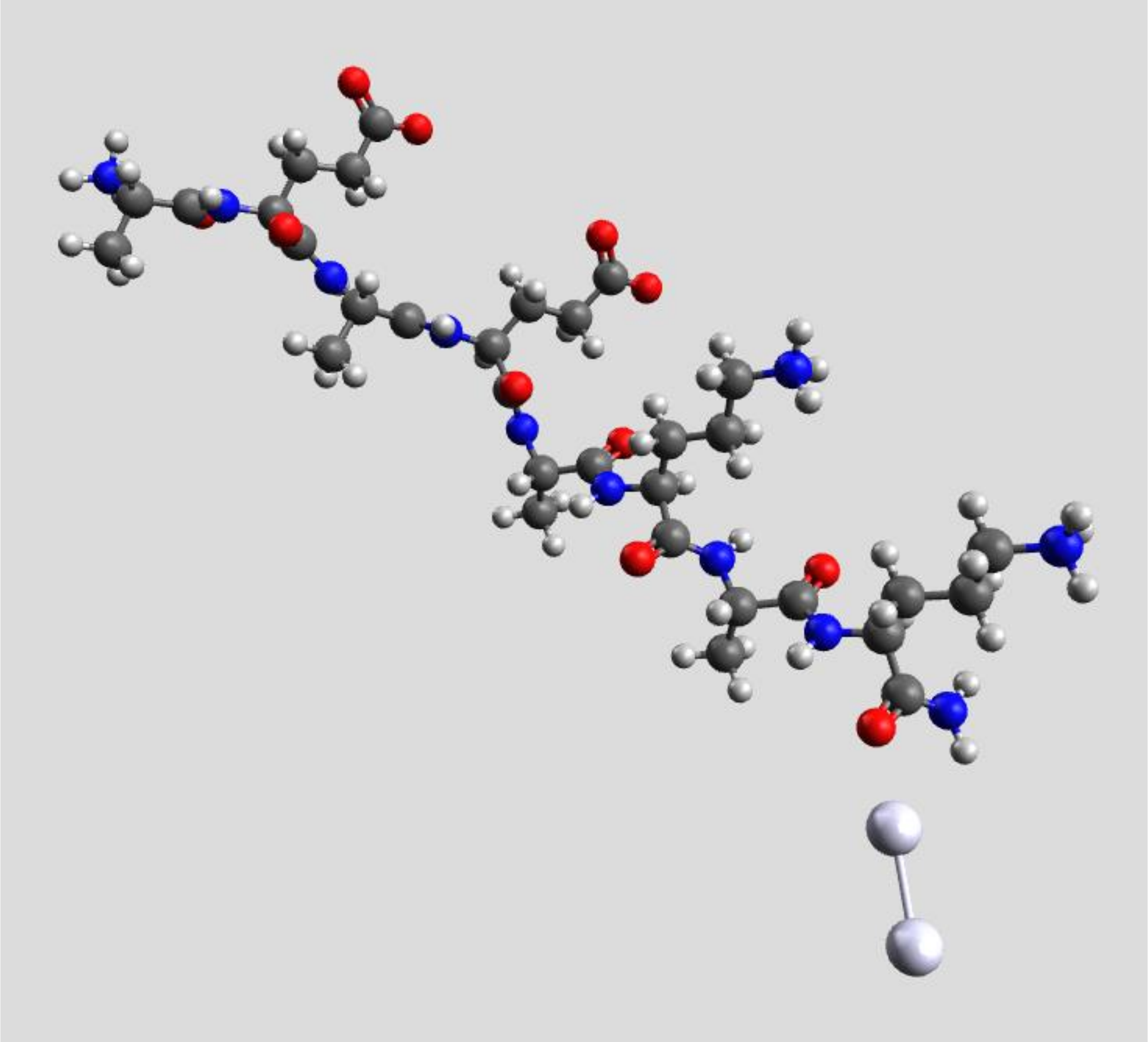

Table reports experimental frequencies (ν_{exp}, cm^{-1}), calculated frequencies (ν_{calc}, cm^{-1}), scaled frequencies ($\nu_{scaled} = 0.9330*\nu_{calc} + 29.97$; scaling straightline), percent experimental Raman Intensities (AR_{exp}), percent theoretical Raman Intensities (AR_{calc}), PED% (sxx-PED%, where sxx is the xxth internal coordinate) and mode Interpretation (ν = stretching, β□= bending, τ = torsion, *out* = out-of-plane). Coalescence of several theoretical frequencies into a scaled frequency is the result of the PED fitting procedure.

ν_{exp}	AR_{exp} %	ν_{scaled}	AR_{calc} %	ν_{calc}	PED%	Interpretation
3071	1.6	2996	3.3	3179	s21-24 s22-74	ν(CH)
		2974	11.4	3157	s51-95	ν(CH)
2973	31.3			3155	s31-95	ν(CH)
				3154	s38-91	ν(CH)

ν_{exp}	AR_{exp} %	ν_{scaled}	AR_{calc} %	ν_{calc}	PED%	Interpretation
		2968	6.1	3149	s19-83 s20-15	ν(CH)
		2982	17.1	3167	s19-15 s20-84	ν(CH)
				3166	s39-94	ν(CH)
				3164	s49-99	ν(CH)
				3162	s29-100	ν(CH)
		2964	4.1	3145	s52-95	ν(CH)
		2955	2.6	3136	s32-80 s33-19	ν(CH)
		3002	5.2	3186	s46-98	ν(CH)
				3184	s57-98	ν(CH)
		2939	20.8	3122	s32-20 s33-68 s35-12	ν(CH)
				3122	s53-92	ν(CH)
				3120	s24-76 s25-11 s26-11	ν(CH)
				3118	s43-11 s47-88	ν(CH)
				3118	s43-85 s47-11	ν(CH)
				3117	s58-98	ν(CH)
		2935	5.2	3113	s48-96	ν(CH)
2936	100.0	2927	26.1	3106	s28-97	ν(CH)
				3105	s55-95	ν(CH)
				3104	s44-88	ν(CH)
		2921	17.2	3099	s24-11 s26-85	ν(CH)
				3099	s33-12 s35-87	ν(CH)
		2948	1.8	3127	s42-89	ν(CH)
		2917	5.8	3095	s37-98	ν(CH)
		2951	2.0	3130	s24-11 s25-85	ν(CH)
		2883	100.0	3063	s54-86 s56-11	ν(CH)
				3063	s41-89	ν(CH)
				3062	s40-95	ν(CH)
				3062	s50-95	ν(CH)
				3060	s30-98	ν(CH)
2874	29.1			3057	s54-12 s56-88	ν(CH)
				3056	s45-92	ν(CH)
		2893	27.8	3070	s21-75 s22-24	ν(CH)
				3069	s34-98	ν(CH)
				3069	s23-97	ν(CH)
2786	1.5	not explained				

ν_{exp}	AR_{exp} %	ν_{scaled}	AR_{calc} %	ν_{calc}	PED%	Interpretation
2737	2.3	out of range				
1676	19.0	not explained				
1638	20.7	1639	7.9	1725	s60-66	ν(OC)
		1636	2.1	1722	s61-64	ν(OC)
		1630	0.5	1715	s65-71	ν(OC)
		1623	0.2	1708	s64-60	ν(OC)
1601	19.4	1602	2.3	1685	s59-73	ν(OC)
		1599	2.9	1681	s63-74	ν(OC)
		1608	0.5	1691	s66-70	ν(OC)
		1614	0.4	1698	s62-66	ν(OC)
		1582	0.6	1663	s127-82 s242-10	β(HNH)+*out*(NHCH)
1549	13.7	1540	2.9	1620	s126-80 s237-14	β(HNH)+*out*(NHCH)
				1618	s71-13 s122-77	ν(NC)+β(HNH)
		1565	0.7	1645	s129-80 s241-13	β(HNH)+*out*(NHCH)
		1568	0.7	1648	s130-84 s244-15	β(HNH)+*out*(NHCH)
		1579	1.1	1660	s132-78 s243-20	β(HNH)+τ(HNCC)
				1660	s124-80 s239-14	β(HNH)+*out*(NHCH)
1502	7.3	1505	0.3	1580	s76-21 s78-13 s120-10 s121-17	ν(NC)+β(HNC)
		1501	0.2	1576	s73-14 s75-18 s116-14 s118-19	ν(NC)+β(HNC)
		1512	1.3	1591	s76-11 s78-18 s120-14 s121-19	ν(NC)+β(HNC)
				1590	s69-15 s74-16 s117-18	ν(OC)+ν(NC)+β(HNC)
				1588	s69-75	ν(OC)
				1588	s67-89	ν(OC)
		1491	1.9	1567	s73-10 s74-12 s75-15 s117-13 s118-20	ν(NC)+β(HNC)
				1564	s77-34 s119-25	ν(NC)+β(HNC)
		1520	0.2	1597	s72-20 s115-34	ν(NC)+β(HNC)
1449	22.5	1445	0.2	1516	s125-92	β(HNH)
		1459	0.1	1531	s128-95	β(HNH)
		1419	3.5	1491	s172-41 s285-10	β(HCH)+τ(HCCC)
				1489	s161-64 s273-25	β(HCH)+τ(HCCH)
				1487	s71-13 s166-10 s172-17	ν(NC)+β(HCC)+β(HCH)
		1467	0.1	1540	s131-96	β(HNH)

ν_{exp}	AR_{exp} %	ν_{scaled}	AR_{calc} %	ν_{calc}	PED%	Interpretation
		1397	4.6	1469	s157-83	β(HCH)
				1467	s168-71	β(HCH)
				1464	s134-75 s249-14	β(HCH)+*out*(CHCH)
				1464	s153-59 s265-20	β(HCH)+ τ(HCCN)
		1386	13.2	1460	s145-52 s257-12	β(HCH)+ τ(HCCN)
				1458	s165-53 s277-19	β(HCH)+ τ(HCCN)
				1456	s152-62	β(HCH)
				1456	s148-61 s267-16	β(HCH)+ τ(HCCN)
1394	37.3			1456	s143-65	β(HCH)
				1455	s163-58 s276-23	β(HCH)+ τ(HCCN)
				1453	s172-10 s282-43	β(HCH)+ τ(HCCN)
		1381	3.6	1449	s170-72	β(HCH)
				1448	s68-39 s101-13 s254-13	ν(OC)+ ν(CC)+ τ(HCCO)
				1448	s70-37 s104-12 s262-27	ν(OC)+ ν(CC)+ τ(HCCO)
		1407	0.6	1476	s136-44 s246-19	β(HCH)+*out*(CCCH)
		1367	1.4	1433	s136-27 s246-28	β(HCH)+*out*(CCCH)
		1376	0.1	1443	s274-54	τ(HCCN)
		1326	1.3	1389	s139-56 s154-14	β(HCH)
				1389	s164-59	β(HCH)
		1318	1.7	1382	s139-18 s151-17 s164-10	β(HCH)+β(HCC)
				1380	s150-12 s261-41	β(HCH)+ τ(HCCC)
		1332	0.6	1396	s139-12 s154-10 s164-11 s275-11	β(HCH)+*out*(CCCH)
				1395	s154-55	β(HCH)
1324	18.0	1341	0.5	1408	s135-87	β(HCH)
				1405	s172-16 s279-22	β(HCH)+ τ(HCCC)
		1355	6.4	1421	s68-25 s141-48	ν(OC)+β(HCH)
				1419	s70-25 s150-42	ν(OC)+β(HCH)
				1418	s155-15 s275-11	β(HCC)+*out*(CCCH)
		1338	0.1	1402	s146-11	β(HCC)
		1350	1.1	1415	s146-10	β(HCC)
		1307	3.8	1371	s171-16 s285-16	β(HCN)+ τ(HCCC)
				1369	s171-35 s284-10 s285-12	β(HCN)+ τ(HCCC)
				1368	s133-27	β(HCC)
1305	15.9	1304	2.0	1365	s160-22	β(HCC)
		1300	1.2	1361	s167-16 s169-16	β(HCC)
		1314	1.3	1376	s141-10 s250-10 s252-19	β(HCH)+ τ(HCCN)+ τ(HCCC)

ν_{exp}	AR_{exp} %	ν_{scaled}	AR_{calc} %	ν_{calc}	PED%	Interpretation
1261	13.5	1293	2.2	1355	s151-10 s268-35	*β*(HCC)+*out*(CCNH)
				1352	s259-13	*τ*(HCCN)
		1288	2.8	1348	s156-11 s275-22	*β*(HCC)+*out*(CCCH)
		1261	1.6	1320	s121-10 s162-21 s166-18	*β*(HNC)+*β*(HCC)
		1268	1.9	1327	s261-13 s264-10	*τ*(HCCC)+*out*(CCNH)
		1256	0.5	1314	s264-26	*out*(CCNH)
		1279	4.7	1341	s250-47	*τ*(HCCN)
				1339	s158-14 s166-10 s169-17	*β*(HCC)
				1338	s158-18 s275-15	*β*(HCC)+*out*(CCCH)
		1284	0.9	1344	s255-48	*out*(CCNH)
1238	13.9	1238	0.7	1295	s268-10 s271-49	*out*(CCNH)+*τ*(HCCC)
		1245	1.2	1302	s281-46	*τ*(HCCN)
		1249	2.7	1307	s140-13 s149-43	*β*(HCC)
				1305	s140-38 s149-16 s253-10 s254-12	*β*(HCC)+*τ*(HCCO)
		1224	4.2	1280	s72-12 s115-21	*ν*(NC)+*β*(HNC)
		1218	2.5	1274	s120-12	*β*(HNC)
				1273	s118-11	*β*(HNC)
		1210	0.4	1265	s147-13 s262-10	*β*(HCC)+*τ*(HCCO)
				1264	s167-22	*β*(HCC)
		1206	0.3	1260	s138-11 s251-14	*β*(HCC)+*τ*(HCCC)
1159	7.7	1159	3.0	1210	s84-17 s265-10	*ν*(NC)+*τ*(HCCN)
		1145	1.8	1197	s79-14 s80-10	*ν*(NC)
				1195	s79-14	*ν*(NC)
		1185	2.9	1238	s77-11 s119-12 s120-19 s155-11	*ν*(NC)+*β*(HNC)+*β*(HCC)
				1236	s133-12 s237-23 s248-14 s249-10	*β*(HCC)+*out*(NHCH)+*out*(CHCH)
		1191	2.9	1246	s119-19	*β*(HNC)
				1244	s116-12 s117-11 s142-11	*β*(HNC)+*β*(HCC)
		1200	2.8	1254	s116-13	*β*(HNC)
				1254	s78-10 s121-15	*ν*(NC)+*β*(HNC)
1126	17.4	1124	2.6	1175		
				1172	s138-33	*β*(HCC)
				1172	s147-10	*β*(HCC)
		1133	0.3	1183		
1104	18.2	1103	2.8	1151	s283-30	*τ*(HCCC)
		1114	0.8	1162	s81-42	*ν*(NC)

ν_{exp}	AR_{exp} %	ν_{scaled}	AR_{calc} %	ν_{calc}	PED%	Interpretation
		1095	0.2	1142	s82-14 s91-15	ν(NC)+ ν(CC)
		1079	3.1	1125	s82-17	ν(NC)
		1087	4.4	1137	s85-15 s270-10	ν(NC)+ τ(HCCC)
				1135	s201-12 s239-17 s249-13	β(CCC)+*out*(NHCH)+*out*(CHCH)
1082	16.6			1132		
				1130	s89-11 s90-12	ν(CC)
		1075	2.0	1120	s89-21 s90-14	ν(CC)
		1069	2.6	1114	s102-25 s336-17	ν(CC)+*out*(CCNC)
				1114	s106-29	ν(CC)
		1053	3.8	1097	s103-35	ν(CC)
				1095	s88-53	ν(CC)
		1045	9.1	1092	s100-62	ν(CC)
				1090	s83-45	ν(CC)
				1088	s86-67	ν(CC)
1051	13.9			1085	s109-19	ν(CC)
		1038	5.2	1081	s109-18	ν(CC)
				1079	s162-24 s276-11	β(HCC)+ τ(HCCN)
		1063	2.0	1108	s71-16 s85-10 s123-20	ν(NC)+β(HNC)
		1033	3.3	1076	s96-32	ν(CC)
				1074	s107-27 s112-18	ν(CC)+ ν(NC)
		1003	0.5	1043	s97-61	ν(NC)
		1007	0.6	1047	s112-53	ν(NC)
1000	4.3	1015	2.3	1056	s91-10	ν(CC)
		1020	1.0	1063	s137-10	β(HCC)
				1060		
		976	0.7	1013	s81-15 s89-15 s239-19 s248-12 s249-12	ν(NC)+ ν(CC)+*out*(NHCH)+*out*(CHCH)
		935	8.4	972	s265-10	τ(HCCN)
				972	s248-15	*out*(CHCH)
				970		
951	18.1			969		
		969	3.1	1008	s244-17	*out*(NHCH)
				1006	s158-10 s241-19	β(HCC)+*out*(NHCH)
		964	0.4	1001	s237-30 s249-18	*out*(NHCH)+*out*(CHCH)
908	16.7	904	6.4	936		
		909	0.1	942	s241-12	*out*(NHCH)

ν_{exp}	AR_{exp} %	ν_{scaled}	AR_{calc} %	ν_{calc}	PED%	Interpretation
		916	0.6	950	s93-11 s174-10	ν(CC)+β(NCO)
		892	6.3	925	s70-12 s104-31	ν(OC)+ ν(CC)
				924	s68-15 s101-39 s181-11	ν(OC)+ ν(CC)+β(OCO)
				923	s106-15	ν(CC)
		921	2.2	955	s87-42 s243-10	ν(CC)+ τ(HNCC)
		924	0.3	959	s111-20 s242-19	ν(CC)+*out*(NHCH)
		871	0.4	902	s102-18	ν(CC)
		863	3.5	893	s108-44	ν(NC)
873	3.8	881	0.3	912		
		853	0.6	882		
		832	0.4	860	s156-10 s272-17	β(HCC)+ τ(HCCN)
		761	1.0	784	s331-35	*out*(NCOC)
				783	s273-10 s330-27 s331-12	τ(HCCH)+*out*(OCNC)+*out*(NCOC)
		769	0.8	792	s327-32	*out*(OCNC)
		755	0.5	777	s328-38	*out*(OCNC)
		748	1.6	771	s326-31 s327-15	*out*(OCNC)
				769	s326-18 s330-11	*out*(OCNC)
763	3.9			768	s325-52	*out*(OCNC)
		740	1.4	762	s322-47	*out*(OCNC)
				760	s323-43	*out*(OCNC)
		794	0.5	819	s99-22	ν(CC)
		733	0.3	753	s280-42 s331-11	τ(HCCN)+*out*(NCOC)
		804	0.2	830	s287-13	*out*(OCOC)
		799	0.1	824	s260-19 s327-11 s329-12	*out*(CCCH)+*out*(OCNC)+*out*(OCOC)
		672	1.3	691	s181-58	β(OCO)
				688	s183-59	β(OCO)
		658	0.3	673	s173-26	β(NCO)
671	1.3	690	0.6	708	s176-12	β(CCO)
				706	s178-26	β(CCO)
		719	2.0	739	s270-15	τ(HCCC)
		716	0.5	735		
640	3.3	648	2.2	662	s177-41	β(NCO)
		631	1.2	644	s175-48	β(NCO)
613	1.9	615	0.5	628	s236-54	τ(HNCC)
		608	0.5	620	s206-11	β(CCN)

ν_{exp}	AR_{exp} %	ν_{scaled}	AR_{calc} %	ν_{calc}	PED%	Interpretation
		625	1.4	638	s179-46	β(CCO)
		598	0.2	608	s236-14	τ(HNCC)
		576	1.7	587	s253-20 s287-25	τ(HCCO)+*out*(OCOC)
				585	s263-38 s329-44	τ(HCCC)+*out*(OCOC)
575	3.2	582	0.3	592	s253-13 s287-17	τ(HCCO)+*out*(OCOC)
		564	0.1	573	s185-14	β(CCN)
		556	0.4	564	s229-72	τ(HNCC)
551	3.6	547	0.2	554	s233-76	τ(HNCC)
		539	0.6	546	s228-81	τ(HNCC)
		535	0.8	542	s230-61	τ(HNCC)
		519	0.3	524	s230-27 s231-11	τ(HNCC)
		512	0.7	517	s232-81	τ(HNCC)
516	2.8	529	0.7	535	s234-82	τ(HNCC)
		504	0.5	508		
		501	0.6	505	s205-28	β(OCO)
		459	0.7	460	s220-41 s333-10	β(CCN)+*out*(CCNC)
		444	0.8	444		
		496	0.5	500	s231-26	τ(HNCC)
466	3.2	492	0.3	496	s231-38	τ(HNCC)
		433	0.8	432	s332-25	*out*(CCNC)
		426	0.8	424	s213-10	β(CCC)
		420	0.5	418	s235-93	τ(HNCC)
		359	1.8	353	s173-11 s221-20 s238-11	β(NCO)+β(CCN)+ τ(HNCC)
				352	s219-51	β(CCN)
		367	2.8	362		
		371	3.0	366	s197-11	β(CCN)
		375	2.5	370		
		340	2.3	332	s240-11	τ(HNCC)
362	2.4	344	0.5	336	s174-16	β(NCO)
		336	0.8	328		
		391	0.9	387	s182-12 s337-15	β(OCO)+*out*(CCNC)
		326	1.4	317	s240-31	τ(HNCC)
		401	0.7	398	s184-12	β(CCO)
		332	0.1	323	s180-10 s240-43	β(NCO)+ τ(HNCC)
		412	0.9	409		

ν_{exp}	AR_{exp} %	ν_{scaled}	AR_{calc} %	ν_{calc}	PED%	Interpretation
		404	0.2	401	s210-33	β(CCC)
